# Signatures of nonsense-mediated mRNA decay but no evidence for transcriptional adaptation associated with protein-truncating variants in wild yeast diploids

**DOI:** 10.1093/molbev/msag219

**Published:** 2026-08-21

**Authors:** Marzena Marszałek, Wiesław Babik, Ryszard Korona, Katarzyna Tomala

**Affiliations:** Institute of Environmental Sciences, Faculty of Biology, Jagiellonian University, 30-387 Kraków, Poland; Doctoral School of Exact and Natural Sciences, Jagiellonian University, 30-348 Kraków, Poland; Institute of Environmental Sciences, Faculty of Biology, Jagiellonian University, 30-387 Kraków, Poland; Institute of Environmental Sciences, Faculty of Biology, Jagiellonian University, 30-387 Kraków, Poland; Institute of Environmental Sciences, Faculty of Biology, Jagiellonian University, 30-387 Kraków, Poland

**Keywords:** genetic compensation, transcriptional adaptation, nonsense-mediated mRNA decay, protein-truncating variants, *Saccharomyces cerevisiae*

## Abstract

Transcriptional adaptation (TA) is a regulatory process in which loss or disruption of gene function caused by protein-truncating variants (PTVs) triggers compensatory changes in expression of the healthy allele or in expression of related genes. Nonsense-mediated mRNA decay (NMD), a conserved RNA surveillance pathway that degrades transcripts containing premature termination codons, is required to initiate this process. In natural yeast populations, PTV mutations occur relatively frequently, raising the question of whether and how their effects are mitigated. In this study, we investigated TA and NMD among PTVs occurring in natural yeast populations. We observed a strong reduction in the abundance of PTV-containing transcripts, suggesting efficient recognition and degradation of transcripts containing premature stop codons. However, despite evidence of this mRNA surveillance activity, we did not detect a clear signature of transcriptional adaptation. While compensation may still occur in specific contexts, particularly for dosage-sensitive genes, it does not appear to represent a general response to PTVs in yeast. The transcriptional deregulation caused by PTVs in natural isolates may be causing too little harm to favor the evolution or maintenance of complex mechanisms required for adequate compensation. The ability to resist specific transcriptomic ruptures would thus rely mostly on the general robustness of genetic networks. Overall, our findings suggest that TA is not a universal response to loss-of-function mutations in yeast.

## Introduction

### Gene expression regulation

Upregulation or downregulation of specific genes plays a major role in shaping phenotypic variation by modulating protein dosage. Such flexibility enables organisms to alter their transcriptome in response to environmental challenges or internal genetic disturbances by adjusting protein production to current demands. While transcriptional responses to external factors are typically shaped by evolution and therefore functionally interpretable, responses to genetic perturbations can be equally complex yet less clearly delineated (De Nadal et al. 2011; Sheltzer et al. 2012; Kovács et al. 2021). In some cases, regulatory mechanisms may act in a compensatory manner, buffering the effects of harmful mutations (McAnally and Yampolsky 2009). However, such compensation requires that the molecular consequences of a disturbance are correctly detected and translated into appropriate changes in gene expression. A key challenge for the cell, therefore, lies in linking the observed phenotypic symptoms of a disturbance to the specific gene(s) whose expression should be adjusted. In principle, a targeted transcriptional response acting on the affected genes would be the most effective and economical strategy. In the real world, however, both detecting the underlying problem and triggering the appropriate regulatory response raise substantial challenges.

From an evolutionary perspective, direct transcriptional adaptation is only one of several strategies that may mitigate the effects of deleterious mutations. In many cases, organisms can tolerate genetic perturbations through alternative mechanisms, such as functional redundancy among genes or buffering via alternative metabolic pathways. (Normanly and Bartelt 1999; Gu et al. 2003). A specific example of gene functional redundancy is transcriptional compensation, a process in which the expression of functionally similar paralogs is regulated to offset the reduced activity or loss of function of another paralog (Kafri et al. 2005; DeLuna et al. 2010; Vande Zande et al. 2023). Another possible mechanism derives from classical genetic theories of dominance within heterozygous loci of diploids (Haldane 1930; Kacser and Burns 1981). When such mechanisms operate in nature, an active transcriptional response to genetic disturbance may not be necessary in certain contexts.

### Protein-truncating variants

Among genetic perturbations, protein-truncating variants (PTVs) are particularly notable because they can be recognized by the cellular quality-control machinery and may therefore trigger specific transcriptional responses. PTVs typically arise from point or frameshift mutations that introduce a premature termination codon (PTC) into the transcript, often resulting in shortened proteins that may be nonfunctional or cytotoxic.

Despite their potentially disruptive nature, PTVs frequently persist in populations (on average, 100 PTVs in a random human genome, MacArthur et al. 2012) and often do not produce an observable phenotype. This apparent tolerance suggests that cellular mechanisms may mitigate their effects, eg, through regulatory responses that compensate for the functional consequences of these mutations. Studies on model organisms—zebrafish, mouse, and *C. elegans*—and humans (El-Brolosy et al. 2019; Ma et al. 2019; Serobyan et al. 2020; Falcucci et al. 2025) have reported compensatory upregulation of related genes (so-called adapting genes) following the introduction of loss-of-function mutations. This phenomenon, often referred to as transcriptional adaptation (TA), involves the upregulation of paralog genes that are sequence-similar to the mutated transcript or upregulation of the wild-type allele of the affected gene.

### Transcriptional adaptation and nonsense-mediated mRNA decay

The first systematic study of transcriptional adaptation, conducted by Rossi et al. (2015), was motivated by phenotypic differences observed between zebrafish egfl7 frameshift mutants and morphants (translation-blocked). Follow-up mechanistic studies demonstrated that transcriptional adaptation is not triggered by loss-of-function mutations per se, but rather by the decay of aberrant mutant mRNA, which acts as the primary signal initiating compensatory gene expression (El-Brolosy et al. 2019; Ma et al. 2019).

In the best-characterized scenario, TA requires the active translation of the defective transcripts, followed by their degradation in the process of nonsense-mediated mRNA decay (NMD) (El-Brolosy et al. 2019; Ma et al. 2019; El-Brolosy et al. 2026). NMD pathway is present in all eukaryotes and relies on the conserved core up-frameshift factors (Upf1, Upf2, Upf3), which are directly associated with the degradation process (Ruiz-Gutierrez et al. 2026).

NMD does not target all defective transcripts equally; some transcripts may escape degradation unless the PTC is recognized as premature. One proposed mechanism of PTC recognition in yeast is the *faux* 3′-UTR model, where an abnormally long distance between the PTC and the normal translation termination promotes transcript degradation (Amrani et al. 2004). Interestingly, beyond aberrant transcripts, NMD also acts on certain normal transcripts that possess features such as long 3′-UTRs or upstream open reading frames (uORFs), thereby contributing to the regulation of gene expression (Celik et al. 2017). Additionally, NMD also targets pervasively translated long noncoding RNAs in yeast (Smith et al. 2014; Malabat et al. 2015; Wery et al. 2016, 2025; Andjus et al. 2024).

Another surveillance mechanism that can contribute to mutant transcript degradation is non-stop decay (NSD) (El-Brolosy et al. 2019). It detects transcripts lacking a proper stop codon and promotes their degradation. In addition to directly degrading non-stop transcripts, NSD has been proposed to contribute to the processing of mRNA fragments generated during NMD by promoting ribosome removal from truncated transcripts (Arribere and Fire 2018). However, unlike NMD, the connection between NSD and TA appears more indirect and has not been mechanistically established.

To date, TA has been found in zebrafish, mouse and human cells, and in *C. elegans* (El-Brolosy et al. 2019; Ma et al. 2019; Serobyan et al. 2020; Falcucci et al. 2025). Those studies focused on a limited number of selected genes in genetically manipulated systems, and only a subset of the mutants examined exhibited compensatory transcriptional responses. More recent work by Mellis et al. (2024) expanded this scope by examining transcriptional adaptation across a broader set of genes. Using the CRISPR-Cas9 transcriptomic dataset in human and mouse cells (74 knockout targets), they demonstrated that TA is a selective and constrained process, with responses varying widely across cells depending on underlying regulatory networks and stochastic effects.

### Gene expression regulation in response to disturbances in yeast

Previous studies in yeast reported that transcriptional responses to diverse environmental stresses are largely global and overlapping across multiple genes (Gasch et al. 2000). Similarly, responses to single-gene deletions are rarely gene-specific but rather rely on functional redundancy, network rewiring, or the accumulation of adaptive mutations to restore fitness partially (Teng et al. 2013; García-Martínez et al. 2021; Kovács et al. 2021). In the case of aneuploidy, results across studies remain inconsistent. Some researchers report little evidence for compensatory regulation, observing that in yeast the expression of most genes scales proportionally with gene copy number (Springer et al. 2010; Torres et al. 2016; Tutaj et al. 2024). In contrast, other studies describe widespread transcriptional dosage compensation on aneuploid chromosomes (Hose et al. 2015; Caudal et al. 2024) or compensation at the protein levels Muenzner et al. (2024). Together, these findings highlight that independent analyses of similar datasets can reach opposing conclusions about compensatory transcriptional responses, underscoring the need for caution in their interpretation. Some research also highlighted differences between laboratory-engineered and natural strains in their responses to aneuploidy, where compensation was observed in natural, but not in laboratory strains (Hose et al. 2015; Muenzner et al. 2024). Collectively, these findings suggest that yeast responses to large-scale genomic perturbations may rely primarily on global, relatively nonspecific buffering mechanisms rather than precise gene-specific compensation, with possible differences between laboratory and natural isolates. This raises the question of whether deleterious mutations, such as PTVs, similarly trigger a global transcriptional response or instead induce gene-specific transcriptional adaptation.

### Knowledge gap

Most studies on transcriptional adaptation have focused on experimentally engineered model organisms, such as zebrafish and mice, and a limited set of genes. However, little is known about this process in *Saccharomyces cerevisiae*. As a simple and exceptionally well-characterized unicellular organism, yeast provides a tractable system for investigating compensatory transcriptional responses across its entire genome-transcriptome system. To the best of our knowledge, no studies have directly tested transcriptional adaptation of PTVs in yeast except for García-Martínez et al. (2021), who examined a single engineered PTC-containing construct by measuring its RNA synthesis rate and found no evidence of compensatory upregulation. Studies in other model systems suggest that TA is not universal across all mutant alleles, implying gene-specific effects and distinct regulatory rules. However, it remains unclear whether TA contributes to the buffering of naturally occurring mutations in wild populations under persistent selective pressure, or how widespread this mechanism is.

Our second focus is NMD, a central driver of transcriptional adaptation that has been well established in yeast, with many of its physiological RNA targets broadly characterized (Celik et al. 2017). However, which PTC-containing transcript variants are recognized and degraded by NMD remains only partially understood, with evidence derived from a limited number of cases.

**Figure 1:**
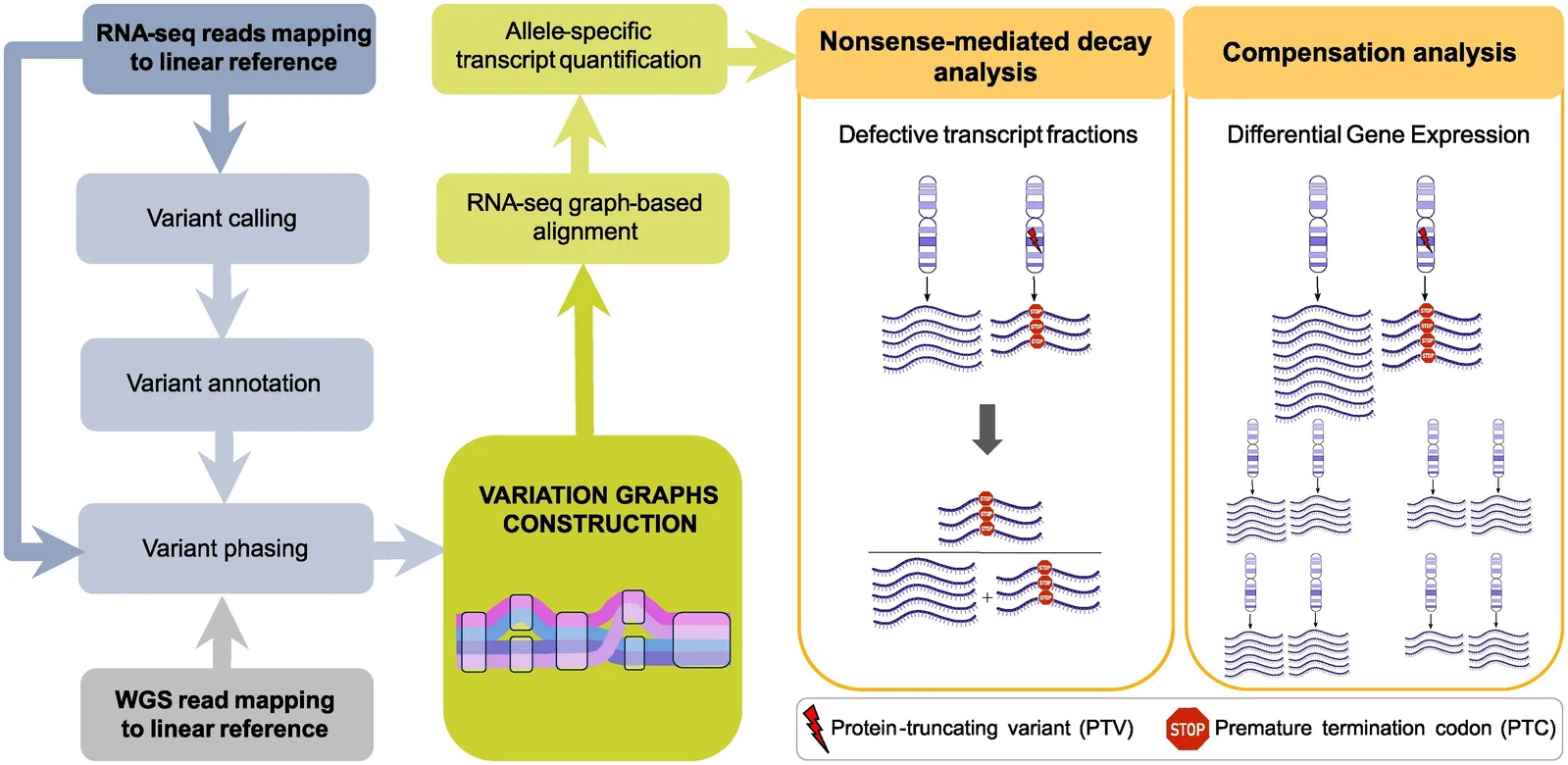
Overview of the analysis workflow. RNA-seq and WGS data were integrated to construct a strain-specific variation graph, which was further applied for allele-specific transcript quantification. Finally, allele-specific counts were used to assess NMD and transcriptional adaptation.

To address these gaps, we integrate RNA-seq (Caudal et al. 2024) and WGS (Peter et al. 2018) data for 282 wild yeast strains to identify reliable protein-truncating variants, for which we perform NMD and transcriptional adaptation (TA) analyses, leveraging graph-based mapping and haplotype-resolved read counting (Fig. 1). Although robust NMD signals were observed across multiple variants, we did not obtain clear evidence for transcriptional adaptation; if present, it appears to be infrequent and potentially restricted to a subset of tightly regulated genes.

## Results

### Abundance and diversity of PTVs

**Figure 2:**
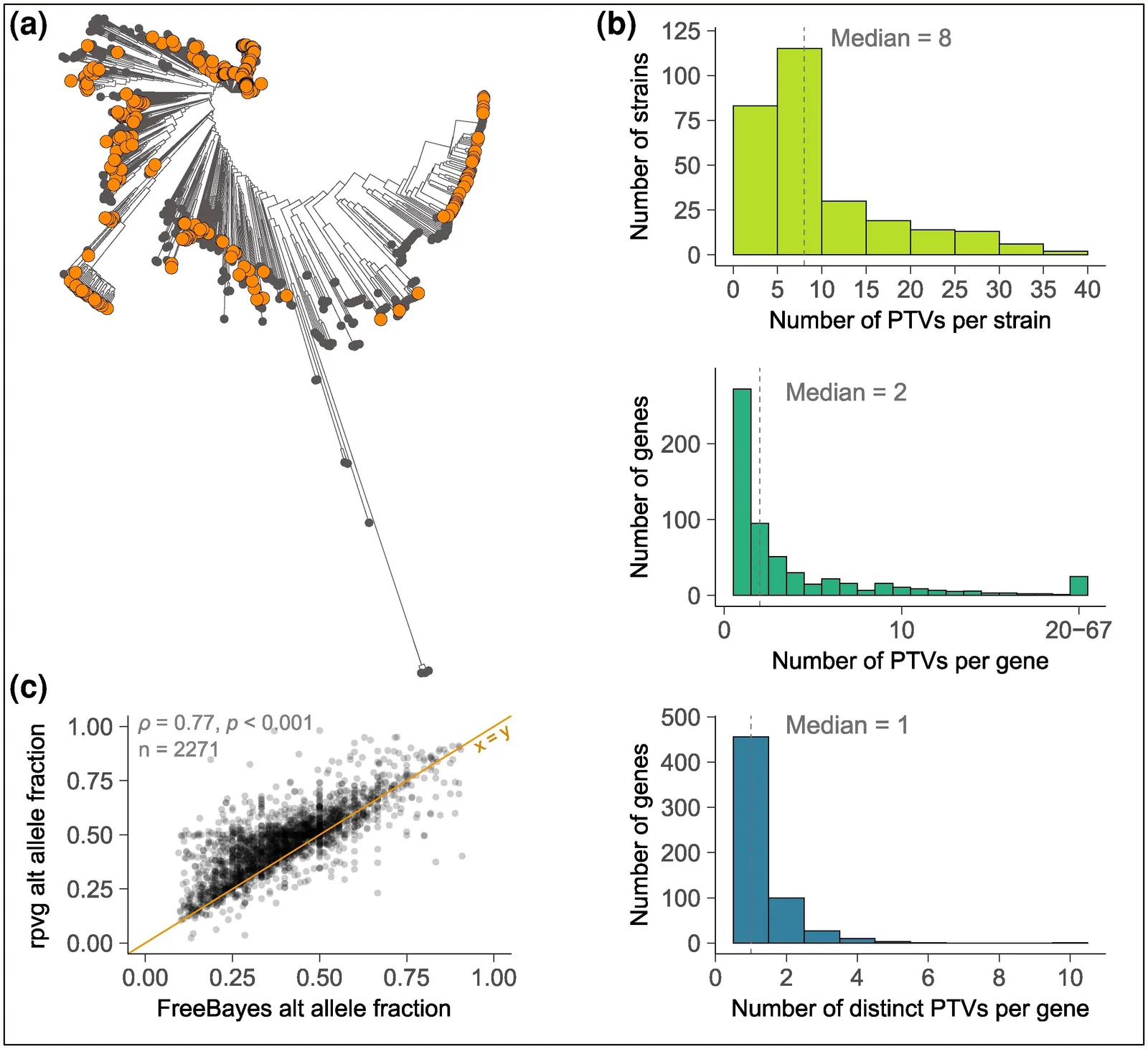
a) Neighbor-joining tree constructed from biallelic SNPs across 1,011 yeast strains (WGS data, Peter et al. 2018). Larger (orange) dots indicate 282 isolates included in this study. b) Summary of identified PTVs: upper panel, number of PTVs per strain; middle panel, gene-level site frequency spectrum for protein-truncating variants (PTVs); lower panel, number of unique PTVs per gene (gene-unique mutations). c) Correlation between alternative allele fractions derived from rpvg and FreeBayes read counts. Spearman correlation is shown for (c).

In this study, we aimed to test whether heterozygous protein-truncating mutations trigger compensatory responses at the transcriptomic level in natural *S. cerevisiae* isolates. The collection of 1,011 natural yeast isolates was genotyped and characterized by Peter et al. (2018), with RNA-seq data subsequently generated by Caudal et al. (2024). For our study, we focused on a subset of this collection that were diploid, heterozygous, euploid strains with RNA-seq data of adequate quality (as described in the Materials and Methods section). Because RNA-seq coverage was moderate, with read counts ranging from 3.9 M to 21.5 M across samples (*mean* = 7.4 M, *median* = 6.9 M), we restricted variant identification to 2,697 genes with moderate-to-high expression levels, for which RNA-seq-based variant calls are expected to be more reliable. Among the 282 selected yeast strains (Fig. 2a), we identified 2,863 heterozygous PTV mutations (Table S2), including 808 distinct PTVs, distributed across 598 genes, ranging from 1 to 37 PTVs per strain. We found 272 genes with a single PTV mutation (mutation in this gene was present in one strain), and around 4% of PTV genes had more than 20 PTVs (ranging from 22 to 67) (Fig. 2b). Overall, the dataset contained a sufficient number of heterozygous PTV mutations to conduct the intended analysis.

Variants included in the analysis were selected based on automated functional annotation (see *Materials and Methods, Variant annotation*). The vast majority were annotated either as stop-gained variants or as frameshift variants with a predicted premature termination codon (ie with an HGVS.p annotation specifying the position of the new stop codon). However, the functional consequences of a rather minor fraction of frameshift variants remain uncertain, as they may instead result in stop-loss, protein extension, loss of productive translation, or translation initiation from an alternative start codon. For simplicity, we refer to all of these variants collectively as PTVs throughout the manuscript, while acknowledging that this classification is an approximation.

### Performance of graph-based vs linear-reference-based PTV quantification

A large fraction of the identified variants are insertions and deletions (INDELs) (1712 INDELs, 1151 SNPs). Such variants may be challenging to align accurately against a linear reference. Moreover, for downstream analyses, we required allele-specific expression (ASE) estimates, but most ASE tools are designed to process only biallelic SNPs, either ignoring INDELs or producing unreliable estimates. To improve mapping efficiency and reduce reference bias, RNA-seq reads were aligned using vg v1.59.0 (Garrison et al. 2018) to a strain-specific variation graph representing both reference and alternative alleles, enabling unbiased mapping of reads from either haplotype. Haplotype-specific expression was then quantified with rpvg (Sibbesen et al. 2023), which leverages the vg alignments to assign and count reads in a haplotype-aware manner. At this stage, we excluded from further analysis PTVs that were not incorporated into the variation graph—typically because they were located near transcript ends—and therefore we could not identify the haplotype carrying the mutation (153 variants), as well as PTVs that were genotyped by rpvg as homozygous (220 variants). We compared graph-based estimates of PTV allele fractions—defined as PTV haplotype expression relative to total gene expression—with estimates derived from Variant Call Format (VCF) data generated using FreeBayes (Fig. 2c), a variant caller operating on reads aligned to a linear reference genome. We found that rpvg estimates were higher than VCF-based estimates for approximately 79% of PTV haplotypes. The 2 methods were strongly correlated (Spearman's *ρ* = 0.77, n = 2,271, *P*  *<* 0.001), while rpvg consistently captured more alternate-allele reads per site (for more comprehensive analyses and detailed results, see *Supplementary Materials, Comparison between rpvg and FreeBayes estimates of allele fractions*). Together, these results suggest that the graph-based approach reduces mapping-related effects, particularly for INDELs, resulting in higher estimated PTV allele fractions compared to the linear-reference approach and more conservative downstream NMD and transcriptional adaptation analyses.

### NMD—the signal in RNA-seq data

**Figure 3:**
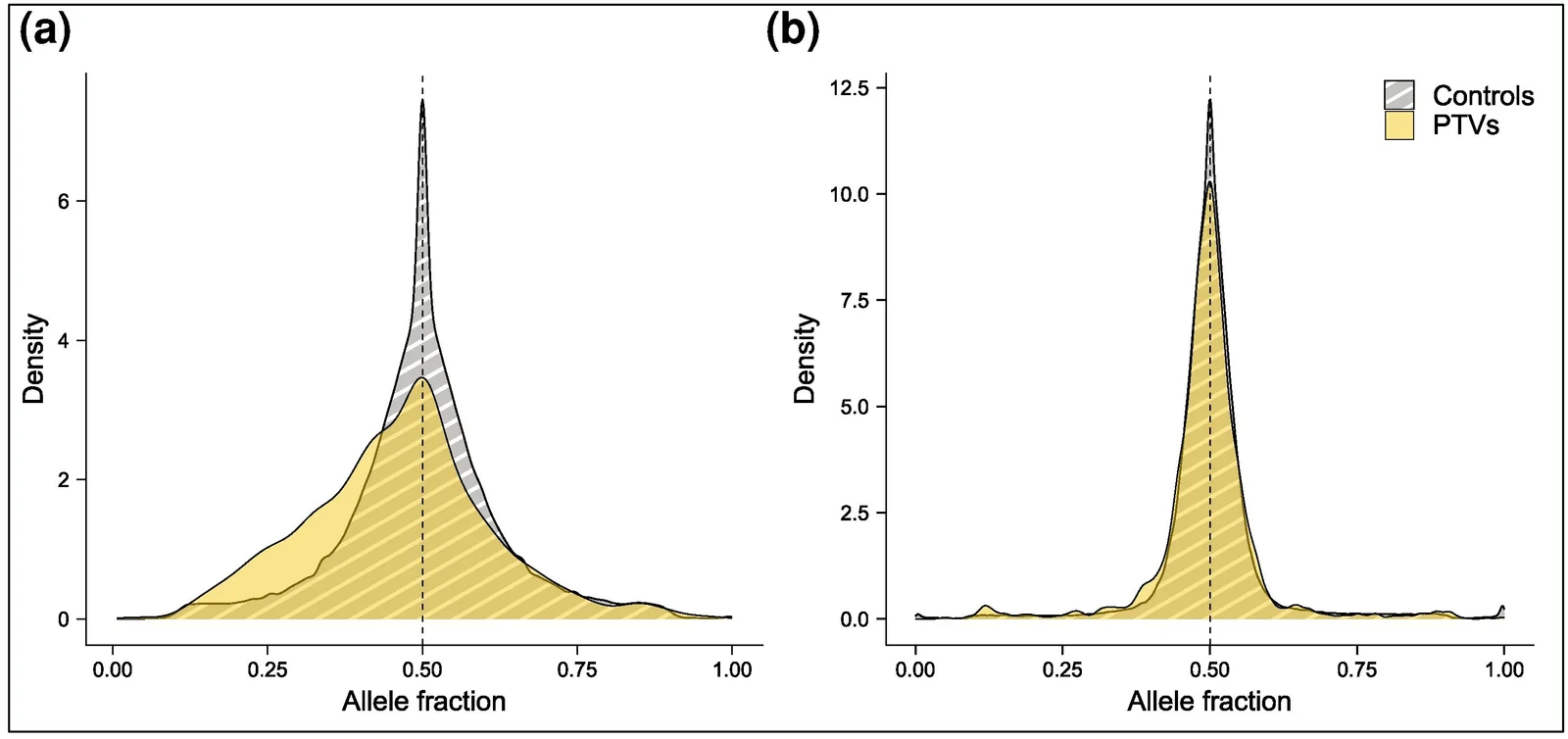
NMD analysis. Density plots showing the fraction of heterozygous PTV alleles (yellow) and the fraction of alleles in PTV-free genes (striped, gray) (see Materials and Methods, *Filtering rpvg output for NMD analyses*). a) Haplotype-specific expression (RNA-seq) and b) Whole-genome sequencing.

Genetic compensation via transcriptional adaptation relies on the presence of the mutated transcript and its degradation, most commonly through NMD, although other RNA surveillance pathways may also be involved (El-Brolosy et al. 2019). We postulated that in heterozygous PTV mutations, degradation of the defective allele may trigger compensatory upregulation of the intact allele of the same gene. Therefore, we first assessed the PTV alleles selected for analysis for indications of NMD-associated behavior. If NMD acts on heterozygous PTV mutations, we would expect decreased contribution of the PTV-bearing haplotype to total gene expression. In line with this expectation, the distribution of haplotype fractions for heterozygous PTV genes was unimodal, with a peak near 0.5, but showed a noticeable skew toward lower values compared to control genes lacking PTV mutations, which exhibited a symmetric distribution (Fig. 3a). The generalized linear mixed-effects (GLMM) analysis was performed on a subset of high-confidence genotypes with a haplotyping probability of 1 (uncertain genotypes excluded), comprising 2271 PTVs and 223,771 controls (genes with heterozygous variants but not PTVs); for the detailed model explanation, see Materials and Methods, *Modeling read count variation by PTV status*. GLMM confirmed that heterozygous PTVs significantly reduce haplotype expression (estimate = −0.16, *P* < 0.001). The effect was largely independent of the strain, as the strain-level random intercept accounted for approximately 13% of the total variation in haplotype fraction (intraclass correlation coefficient, ICC ≈ 0.13). Examination of the random effects identified a single strain with the most extreme deviation (*z* = −14.14). After excluding this strain from analysis (keeping the model unchanged), the ICC dropped to 0.03, indicating that most variation occurs within rather than between strains. Collectively, the fraction density plot and GLMM results indicate that PTC-bearing haplotypes have a lower allelic fraction than other non-PTV heterozygous variants.

### NMD—WGS control

Variants used as controls in NMD testing were predominantly SNPs, whereas PTVs included a substantial proportion of INDELs. To test for mapping bias as a confounding factor, we replicated the analysis of PTV haplotype fractions using whole-genome sequencing data, providing a genomic control for RNA-seq–based effects. The density plot (Fig. 3b) shows that allele fractions for both PTVs and control genes are symmetrically centered around 0.5, as expected for diploid strains without aneuploidies. In contrast, RNA-seq–based fraction distributions (Fig. 3a) are broader, which may reflect an increased imbalance in heterozygous allelic expression. GLMM analysis of WGS fractions (2377 PTVs and 228,352 control genes) revealed a weak effect of PTVs on haplotype representation (estimate = −0.05, *P* < 0.001). This effect might in principle reflect residual mapping bias or, alternatively, subtle loss of heterozygosity (LOH) affecting the mutant allele during strain propagation. The latter class of events could contribute to the observed difference if loss of the PTV-bearing allele confers a fitness advantage. The strain-level variation was substantial (ICC ≈ 0.70). However, the random-effects analysis revealed that the effect was driven by a small number of outlier strains with extremely low heterozygosity; among the 16 most extreme strains, there were only 4–14 control heterozygous genes per strain. After excluding these strains from GLMM (with the model otherwise unchanged), strain-level variation decreased markedly (ICC ≈ 0.02), while the PTV effect decreased further (estimate = −0.03, *P* = 0.001). Thus, excluding the outliers does make the effect for DNA data even smaller. Together, these results indicate that PTV-associated reduction in haplotype fraction is not explained by mapping bias but must be driven by RNA-level processes, consistent with the action of NMD.

### Features associated with the NMD signal

**Figure 4:**
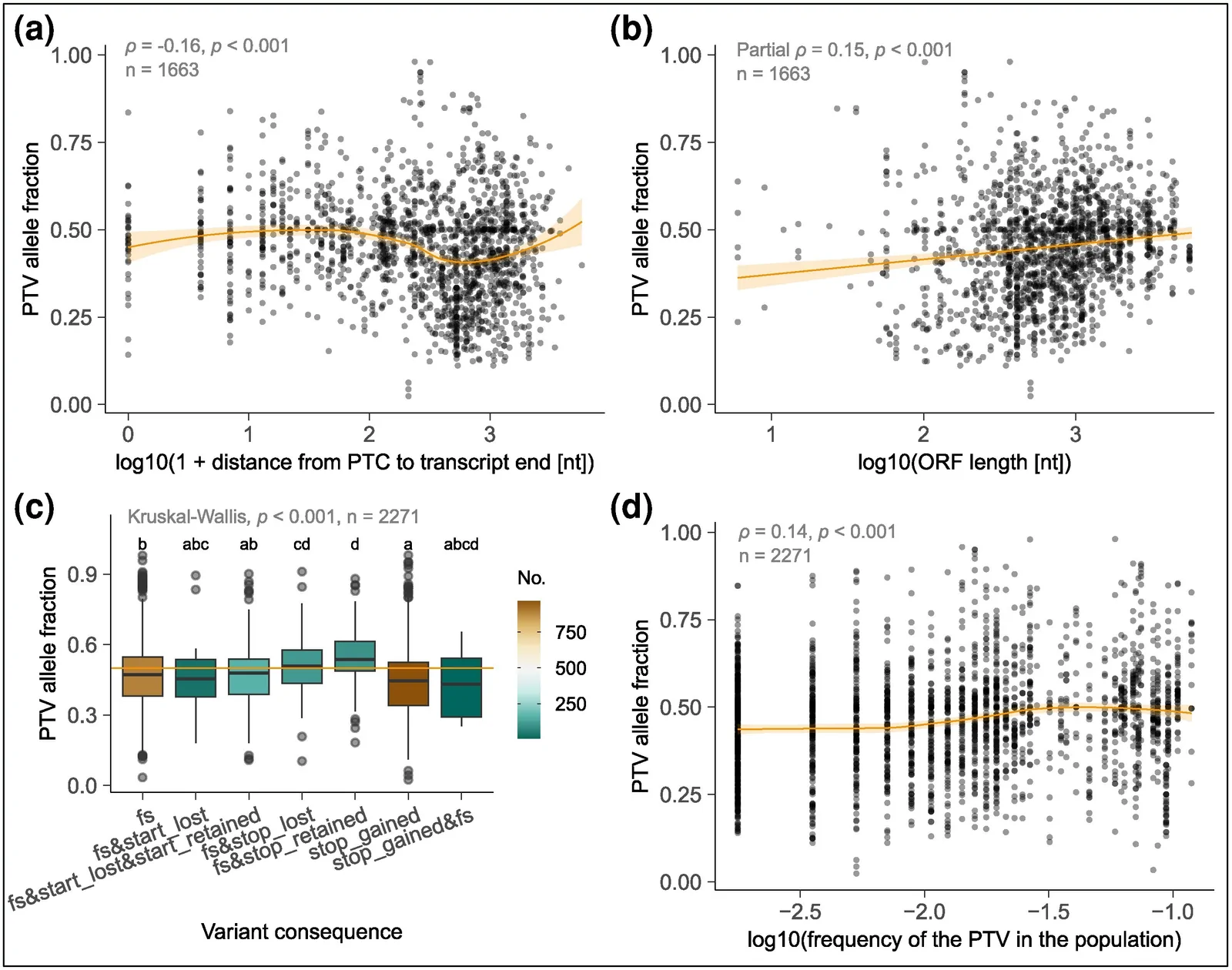
Features associated with NMD. Relationship between the fraction of the PTV allele and a) the distance between PTC and transcript end, b) ORF length, c) PTV mutation type based on VEP consequences (fs denotes frameshift mutation, orange line indicates the fraction of 0.5); categories differ in the certainty of premature codon insertion (the most certain are stop_gained and fs&stop_gained; for further explanation see *Supplementary Materials*), and d) allele frequency in the population. For the analyses presented in (a) and (b), only variants with a known premature stop codon position were included. Fitted lines represent LOESS smooths in (a) and (d). In b), the line represents predictions from a linear model that controls for 3′ UTR length. Shaded regions around lines represent 95% confidence intervals. Spearman correlation coefficient (*ρ*) is shown for (a) and (d), and the partial Spearman correlation is shown for (b). In (c), a–d letters indicate significant differences from the pairwise Wilcoxon test; groups sharing the same letter are not significantly different (*P* > 0.05).

NMD targets mostly PTC-bearing transcripts. In yeast, recognition of stop codons as premature depends largely on their distance from the 3′ end of the transcript (Amrani et al. 2004). PTC located further from the 3′ end creates an abnormally long 3′ UTR, which impairs proper translation termination, increasing the probability of NMD activation. Consistent with this model, we observed a moderate but statistically significant negative correlation between the PTV haplotype fraction and the PTC position (Spearman's *ρ* = −0.16, *n* = 1,663, *P* < 0.001) (Fig. 4a), indicating that variants introducing earlier stop codons tend to be associated with lower haplotype expression. Recent studies have suggested that this apparent progressive relationship may in fact reflect a threshold effect, whereby NMD sensitivity is largely determined by whether the 3′ UTR exceeds approximately 300 nt rather than increasing continuously with 3′ UTR length (Kebaara and Atkin 2009; Andjus et al. 2024). In line with this interpretation, stratification of our data using this threshold revealed that the overall negative correlation observed in the full dataset was no longer present in either subgroup *(Supplementary Materials*, *Nonlinear relationship between NMD efficiency and 3′ UTR length*). Consistently, transcripts with shorter 3′ UTRs exhibited PTV haplotype fractions approaching 0.5. Additionally, Decourty et al. (2014) showed that the length of the truncated open reading frame (ORF) itself, independent of 3′ UTR length, influences NMD sensitivity, with longer ORFs being more likely to escape NMD. Using partial Spearman correlation controlling for PTC-to-end distance, we found statistical support for the expected positive correlation between PTV fraction and ORF length (Partial Spearman's *ρ* = 0.15, *n* = 1,663, *P* < 0.001) (Fig. 4b).

The identified PTVs can be classified into 7 consequence categories based on Variant Effect Predictor (VEP) annotations (Fig. 4c). We compared the NMD signal across these categories and observed significant differences in the fraction of the PTV allele (Kruskal–Wallis *χ^2^* = 96.93, df = 6, *P* < 0.001) (Fig. 4c). Stop-gained variants exhibited the lowest fraction of PTVs, consistent with their role as canonical substrates for NMD. Frameshift variants displayed greater variability, which may reflect differences in their downstream consequences, including whether a premature termination codon is introduced or whether the altered reading frame extends toward the 3′ end of the transcript without encountering a stop codon. In the latter case, transcripts may be subject to non-stop decay, which is also informative for our analyses. Frameshift variants annotated as stop-retained showed the highest fractions. These variants might not introduce PTC and, therefore, might not trigger NMD. Notably, the number of variants in this category was small (*n* = 99). Overall, most variant categories showed a median fraction below 0.5 (Figure 4c), suggesting preferential depletion of transcripts carrying the mutant allele. Moreover, the lowest median fractions were observed for variant categories in which the introduction of a premature termination codon could be inferred with the greatest confidence.

Finally, we examined whether the PTV allele fraction is connected to population allele frequency. We observed a significant positive relationship (Spearman's *ρ* = 0.16, *n* = 2,271, *P* < 0.001) (Fig. 4d), indicating that more common PTV alleles tend to exhibit lower NMD efficiency (higher expression levels). This suggests that common PTV alleles—likely under weaker negative selection—may include variants that do not fully trigger NMD. Notably, 62 PTVs with the highest allele frequencies (0.105–0.119) were annotated as frameshift & stop-retained variants. These might be misclassified as true loss-of-function, which may explain their high frequency.

We are aware that allelic imbalance can arise from various factors, such as unequal expression. However, the most robust explanation for the systematic reduction of the PTV-carrying allele appears to be the NMD activity. The observed association between NMD-related patterns and both PTC distance to the transcript end (3′UTR length) and ORF length is consistent with previous experimental studies (Amrani et al. 2004; Decourty et al. 2014). Moreover, apparent NMD-related effects vary by PTV mutation type and are stronger in rare variants, which are likely subject to stronger purifying selection.

### Heterozygous PTV mutations are associated with changes in transcript abundance

**Figure 5:**
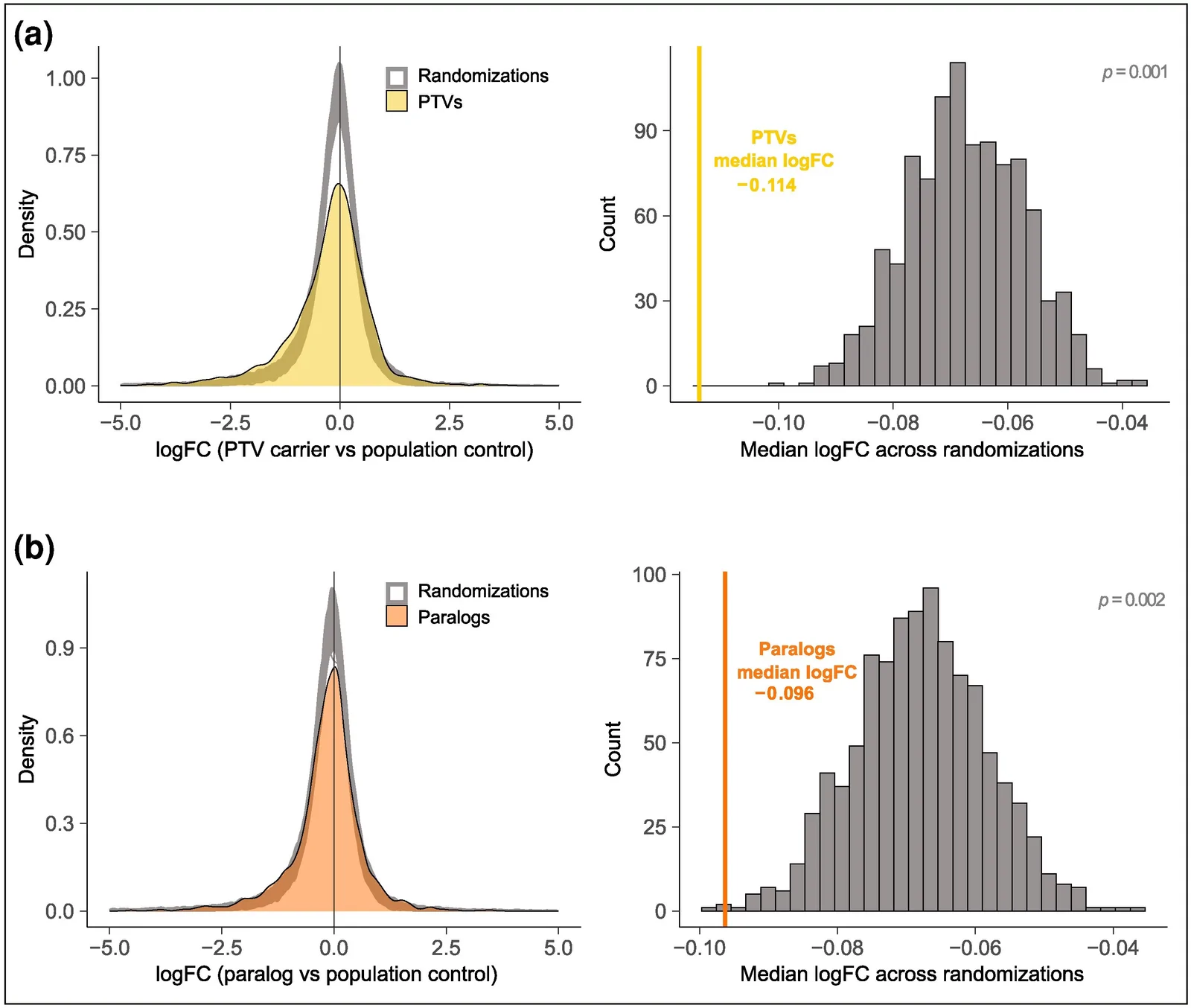
Expression compensation analysis. Density plots illustrate logFC (log fold change) in gene expression between the studied group and the control for: a) genes harboring a heterozygous PTV (wild-type allele expression) and b) paralogs of genes from (a), i.e. paralogs of genes carrying a heterozygous PTV. In (a), both the studied group and the control are defined at the haplotype level (allele-specific expression), whereas in (b), both are defined at the gene level. In each case, the control consists of the expression levels of the same genes across population of all strains, excluding those strains that harbor PTVs or CNVs in those genes. Gray density curves represent logFC distributions from 1,000 randomization tests using randomly selected genes matched to the level of analysis (haplotype-level for a; gene-level for b). Histograms on the right side of each panel show the distribution of median logFC values across randomizations, with the observed median (PTV genes or paralogs) highlighted and an empirical *P*-value indicating its significance relative to the null distribution. For visualization purposes, logFC values were truncated to the range −5 to 5 due to long-tailed distributions.

The main objective of this study was to determine whether PTVs induce genetic compensation via transcriptional adaptation. Since we investigated heterozygous PTV mutations, potential compensatory mechanisms may occur through upregulation of the affected gene—manifested as increased expression of the healthy allele—or related genes, such as functional paralogs. To address this, we analyzed each heterozygous PTV by comparing steady-state transcript levels of the wild-type allele—unaffected by NMD and thus suitable for disentangling degradation and compensatory upregulation—in the variant-carrying strain against those of non-PTV alleles of the same gene across the population (controls: all strains lacking a PTV in that gene). Transcript abundance was quantified as normalized, allele-specific (per-haplotype) counts using rpvg. We further excluded from the control group any strains harboring copy number variation in the same gene. This approach produced a distribution of log fold-change (logFC) values (Table S3). For paralogs, a similar test was performed using gene-level read counts (see Materials and Methods, *Differential gene expression analysis*). We began by analyzing the distribution of expression changes in PTV heterozygotes. The distribution was unimodal with the mode centered near zero, indicating no change (Fig. 5a). If genetic compensation were a common response to PTVs, we would expect a rightward shift reflecting increased expression of the healthy allele. Contrary to this expectation, we observed a modest but consistent shift to the left. A similar pattern was observed for paralogs, although the change was weaker (Fig. 5b). To ensure that the observed pattern was not driven by technical factors (eg the differential gene expression (DGE) method) or by population structure, we performed a randomization-based empirical test with 1,000 iterations (see Materials and Methods, *Randomization test*). In each iteration, we randomly selected a set of non-PTV genes and, for each gene, a single allele whose expression was compared against alleles from the population. To mirror the design of the main analysis, where the number of PTVs tested and the number of control strains varied by gene, we adjusted these numbers accordingly. For each iteration, we recorded the median of the resulting logFC distribution (null distribution), which was then compared to the median obtained for the true PTV group. The null distributions showed a slight shift toward negative values rather than being centered at zero, potentially reflecting technical factors or the nonuniform genetic relatedness among the strains, which may result in genome-wide shifts in expression differences. However, the median logFC of PTVs (median = −0.114) and their paralogs (median = −0.096) were significantly lower than that of 1,000 randomly sampled gene sets (*P* < 0.001 for PTVs, and *P* = 0.002 for paralogs) (Fig. 5a and b, right panels).

**Figure 6:**
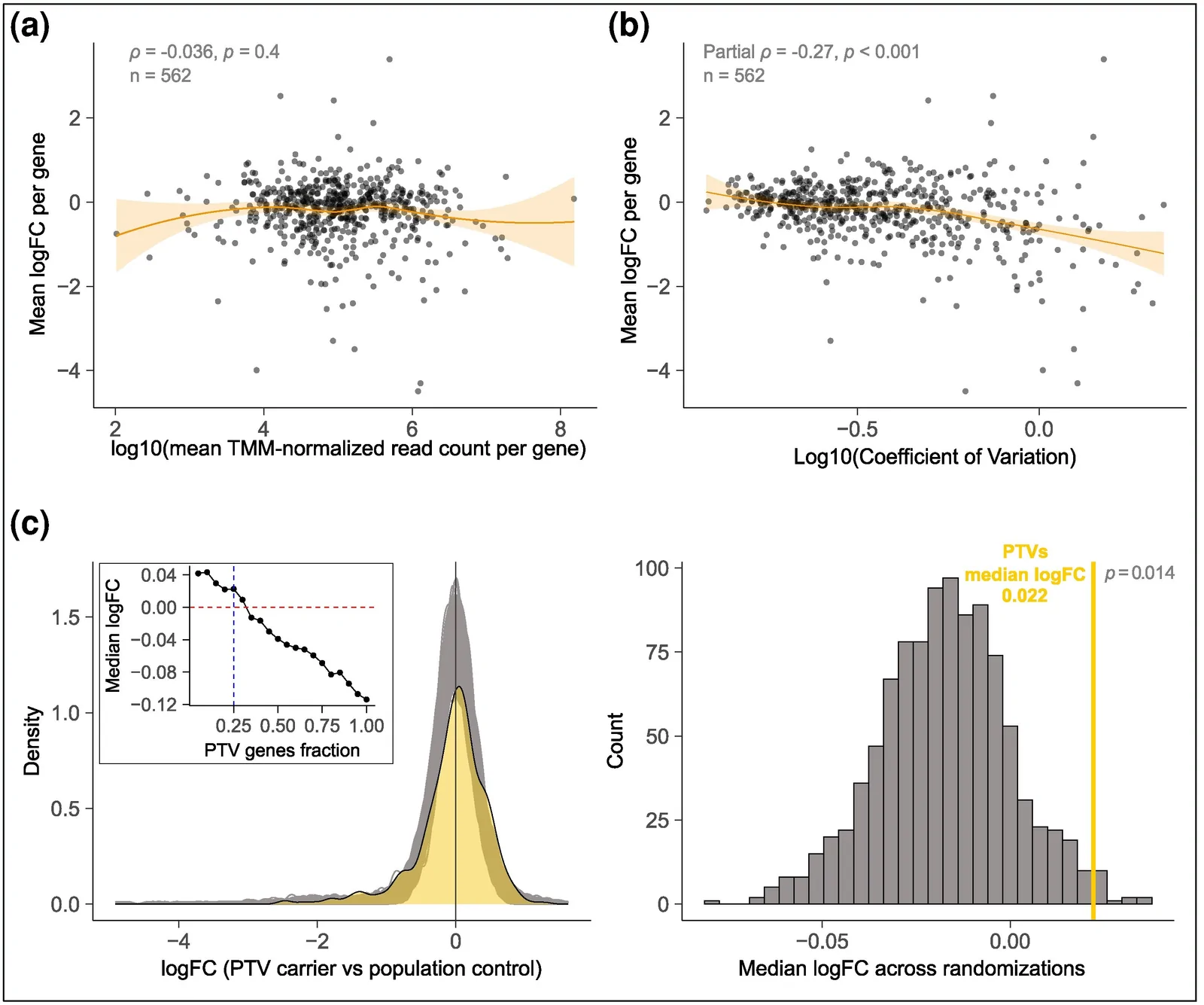
Correlation between mean logFC per PTV-containing gene and a) mean gene expression (across all strains) and b) coefficient of variation, calculated from TMM-normalized read counts. Fitted lines represent LOESS smooths, with shaded regions around lines representing 95% confidence intervals. Spearman correlation coefficient (*ρ*) is shown for (a), and the partial Spearman correlation is shown for (b). (c) A density plot illustrates logFC (log fold change) in gene expression between the studied group and the control for the subset of PTV genes (see Fig. 5a) comprising the 25% of these genes with the lowest coefficient of variation (CV). An additional scatter plot shows logFC as a function of the proportion of genes ranked from lowest to highest expression variability (CV), sampled in 5% increments. The horizontal line indicates logFC = 0 (boundary between reduced expression and compensation), while the vertical line marks the 25% threshold used to define the low-CV subset. For the detailed description of the density plot and histogram, see the Fig. 5 caption.

The more pronounced leftward shift in PTVs expression changes may suggest an adaptive response aimed at reducing the abundance of damaged transcripts and, therefore, possibly cytotoxic proteins. This response would be expected to be more pronounced for highly expressed proteins, since the deleterious effect should scale with the number of damaged protein molecules produced in the cell. Contrary to this expectation, we found no correlation between gene expression and logFC in PTV-containing genes (Spearman's *ρ* = −0.036, *n* = 562, *P* = 0.4) (Fig. 6a). An alternative explanation is that PTVs are more frequently observed in genes of lower functional importance in strains carrying these variants, as weaker purifying selection allows them to persist. Consequently, reduced expression of genes harboring PTVs would be expected to be more frequent among genes with high population-level variability in expression—an indicator of weaker regulatory control. Consistent with this interpretation, genes with higher expression variability (CV)—an indicator of weaker regulatory control—exhibited larger negative expression changes, with the mean downward shift of expression being significantly correlated with CV (Spearman's *ρ* = −0.268, *n* = 562, *P* < 0.001) (Fig. 6b). Importantly, this relationship remained significant after controlling for expression level (Partial Spearman's *ρ* = −0.266, *n* = 562, *P* < 0.001), indicating that the effect is not driven by mean gene expression. Together, these measures suggest that genes of low functional importance can undergo decreased expression, minimizing the production of defective proteins. In contrast, genes under stronger selection compensate by maintaining or increasing expression, even at the cost of producing some defective proteins. Overall, the expectation that transcriptional adaptation would lead to a universal increase in abundance of the healthy variant appears overly simplistic when applied to a limited phenomenon in wild yeast strains, in which some genes (functions) may not be exposed to selection strong or long enough to promote such adjustments.

### Rare cases of allele-specific up-regulation in heterozygous PTVs

Genes with lower expression variability are likely dosage-sensitive and thus subject to stronger selective constraint. To test this hypothesis, we restricted the compensation analysis to a sample of 141 genes (representing one-quarter of all PTV-containing genes) with the lowest coefficients of variation, as potentially the most likely to exhibit compensatory responses. Accordingly, differential gene expression (DGE) results were limited to this subset, and the number of genes tested per strain in each of the 1,000 random DGE draws was adjusted. After filtering, 466 PTVs across 209 strains remained for analysis.

The distribution of logFC values showed now a slight shift toward higher expression (Fig. 6c, left panel), with a median of 0.02. A randomization test confirmed the significance of this pattern (*P* = 0.014). We quantified the increase in expression relative to the null expectation as the difference in median logFC between the true PTV set and the null distribution (*Δ*), resulting in a fold change of 2^*Δ* (Fig. 6c, right panel). Although statistically significant, the estimated increase was small (2.7%). Within this PTV subset, no cases exhibited both significant NMD and at least a 2-fold upregulation, whereas only 8 such cases were identified across all PTVs. Although the 25% threshold was defined a priori, inspection of the full CV range revealed a monotonic shift in median logFC, transitioning from positive to negative values at a cutoff corresponding to approximately the lowest 30% of genes by coefficient of variation (Fig. 6c). Overall, we detected a small yet statistically significant increase in expression among genes with low variability in expression across strains.

## Discussion

### PTVs in the natural yeast strains

The yeast data used in this study (Caudal et al. 2024) come from natural populations (Peter et al. 2018) across 5 continents and diverse ecological niches. Genetic variation in these populations has likely been shaped by extended evolutionary processes. These include mutation, genetic drift, and natural selection acting over many generations. In particular, natural selection can act on gene expression levels, which in turn influence phenotypic traits. Accordingly, the observed expression patterns may reflect a combination of molecular regulatory mechanisms and population-level evolutionary history. We believe that the set of identified PTV mutations, with both frequent and rare ones, likely represents (i) a combination of variants having mild or neutral functional consequences in a certain strain/environment context, and (ii) recently arisen ones that have not yet been eliminated by purifying selection. This should allow for the statistical identification of recurrent gene expression responses to loss-of-function mutations across multiple genes, including both immediate reactions to genetic disruption and slower adaptive processes over time.

### Heterozygous PTV allele fraction as the indicator of NMD

We identified a group of heterozygous PTV genes with a lower proportion of the PTV allele in RNA-seq reads. This pattern could arise from several factors, including allele-specific expression and technical biases during sequencing, PCR, or read mapping. However, we interpret this pattern as the result of primarily NMD, as it is the main known mechanism that directionally reduces the abundance of transcripts from PTV-containing alleles across multiple genes. Other mechanisms, except for mapping bias, are expected to produce deviations in both directions. We can effectively rule out mapping bias as a significant factor because a similarly low proportion of the PTV allele was not observed in the WGS data. We also observed a moderate but statistically significant correlation between NMD efficiency and both PTC-to-transcript-end distance and ORF length, with a weaker NMD-associated pattern in longer ORFs and when PTCs are closer to the transcript end, consistent with previous studies (Amrani et al. 2004; Decourty et al. 2014). To the best of our knowledge, this is the first study suggesting that NMD may affect such a broad set of PTV-containing genes in yeast. While similar analyses have been performed in humans (Rivas et al. 2015; Teran et al. 2021), evidence from a species with a distinct NMD mechanism provides complementary insight into the general principles governing this process.

### PTV gene downregulation may help to mitigate deleterious mutation effects

The paucity of potential transcriptional adaptation among heterozygous PTV-containing genes and their paralogs suggests that gene-specific compensatory upregulation is not a widespread response in natural yeast populations. Although we observed slight upregulation in a subset of PTV genes, the effect was generally small. Instead, our observations indicate that transcriptional suppression is a more common response to deleterious mutations. We propose that this suppression may be adaptive, as it could reduce the production of dysfunctional and potentially deleterious protein products. Additionally, this pattern may reflect coordinated regulation at the pathway level: reduced activity of a defective gene could decrease flux through its associated biological pathway, triggering the downregulation of functionally related genes. This mechanism could also explain the observed downregulation of paralogs, consistent with a broader systems-level response rather than gene-specific compensation. Alternatively, this pattern may not be adaptive but may instead reflect relaxed selective constraint, with PTVs preferentially occurring in genes and pathways of lower functional importance in certain strains. The lack of widespread upregulation of paralog genes also suggests that we did not detect evidence of the previously described functional transcriptional compensation between them (Kafri et al. 2005; DeLuna et al. 2010; Vande Zande et al. 2023). This is, however, consistent with other earlier studies showing that transcriptional compensation is restricted to a small subset of closely related paralogs with highly conserved functions, is often unidirectional, and has primarily been demonstrated in haploid systems, where gene loss represents a much stronger perturbation than heterozygous gene disruption (DeLuna et al. 2010).

### Three potential explanations for the paucity of transcriptional adaptation signal in yeast: technical, mechanistic, and evolutionary

Our analyses proved to be technically challenging. We aimed to assemble a robust pipeline using the most appropriate available tools for our data; however, even the best tools have their limitations. We applied rpvg, a pangenome-based transcript quantification method, which requires phased variants. Unfortunately, the phase of a substantial fraction of our variants could not be resolved. To mitigate potential effects of phase switching on read quantification, we applied rigorous filtering to variants intended for integration into the variation graph (Materials and Methods, *Filtering VCF files based on phasing quality*). The resulting alternative allele fractions correlate well with those generated using FreeBayes, indicating that, despite minor phasing ambiguities, the approach yields reliable results. We propose that longer reads and paired-end sequencing could enhance phasing resolution, resulting in more precise quantification. A further potential source of bias is data normalization. We used the TMM (trimmed mean of *M*-values) method, a widely adopted framework for comparing expression across samples. This method assumes that the majority of genes are not differentially expressed (Robinson and Oshlack 2010; Evans et al. 2018), making our approach conservative. Another limitation relates to variant annotation. While we classified variants as putative loss-of-function based on VEP predictions, this group may also include a minor fraction of variants with more complex or less certain consequences, and their functional impact cannot always be straightforwardly inferred from annotation alone. In particular, for some frameshift variants, downstream effects may include protein elongation, loss of a canonical stop codon, disruption of translation initiation or, in some cases, complete loss of productive translation. Notably, stop-retained variants may not alter the encoded protein and may still produce a functional product; however, these were rare in our dataset. Finally, due to the moderate coverage of the available data, our analyses exclude genes with low expression levels. Therefore, if compensatory responses are more likely to occur in such genes, they may have been missed. However, the lack of correlation between mean population-level gene expression and compensation suggests that this limitation is unlikely to affect our conclusion substantially. Taken together, these limitations are expected to primarily reduce statistical power and attenuate detectable signals, while introducing only limited, likely not systematic biases that would materially affect the main conclusions.

The second class of explanations why gene-specific transcriptional adaptation (TA) in yeast is weak may be that the required molecular machinery is incomplete. TA has been observed in metazoan model organisms such as zebrafish and *C. elegans* (El-Brolosy et al. 2019; Serobyan et al. 2020), but no study has yet identified this mechanism in unicellular organisms. TA depends on the transport of a signal from the cytoplasm to the nucleus. In mammalian cells, El-Brolosy et al. (2026) proposed a mechanism in which ILF3 (Interleukin Enhancer Binding Factor 3) mediates the translocation of cytoplasmic NMD intermediates to adapting gene loci in the nucleus. On the other hand, *C. elegans* lacks a direct ILF3 homolog, yet TA occurs through an alternative pathway involving small RNAs (sRNAs) generated from mutant mRNA degradation. These sRNAs are transported into the nucleus by argonaute proteins, including NRDE-3, and can modulate transcription at adapting genes. Yeast, however, lacks both metazoan toll-kits, and to date, no alternative mechanism has been proposed.

It may also be relevant that mRNA degradation via NMD, which precedes any transcriptional adaptation, employs different mechanisms across organisms. In metazoans, the core NMD pathway relies primarily on SMG6-mediated endonucleolytic cleavage of PTC-bearing transcripts, in contrast to yeast, where decapping is followed by 5′-to-3′ exonucleolytic degradation by the Xrn1 enzyme (He and Jacobson 2015). As a result, the yeast cell may not generate sufficiently stable NMD intermediates, since transcripts are degraded progressively, nucleotide-by-nucleotide, further disfavoring ascendance of transcriptional adaptation.

Last but not least, the selective pressure may be generally too weak to favor the evolution of the TA in most yeast genes. Considering a single locus, it appears natural to expect that any mechanism(s) enabling the detection of a damaged allele followed by upregulation of its healthy counterpart would be of clear value. This may be much less obvious if the functioning of an entire genetic network is considered. The ability to detect and react to perturbations in the dose of a single gene transcript would mean that the whole system is highly sensitive to dysregulation among its many constituents. However, the genetic network might effectively buffer expression changes resulting from loss-of-function mutations, limiting their impact at the phenotypic level. Indeed, it has been postulated that the evolution of the transcriptome is mostly neutral (Khaitovich et al. 2004). In the case of yeast, support for this claim has been found when neither intra- nor interspecific variation of gene expression could be explained by the adaptation to a common environment or shared phylogeny (Yang et al. 2017). Although strict neutrality is unlikely, and expression of many genes is thought to evolve under stabilizing selection (Hill et al. 2021; Siddiq and Wittkopp 2022), it is possible that selection might only detect variants with strong effects (i.e. greater than 2-fold decrease in expression). The neutrality of relatively small shifts in gene expression level in a large fraction of genes could explain why we see strong evidence of NMD but only scant indication of transcriptional adaptation. The presence of mutated variants does have a negative impact on cell functioning, but the pressure to evolve a transcriptional antidote may be too weak except for a small fraction of genes with the highest regulatory requirements.

## Materials and methods

### Yeast strain selection

The raw RNA sequencing (RNA-seq) data from Caudal et al. (2024) were downloaded from the European Nucleotide Archive (accession no. PRJEB52153). Whole-genome sequencing (WGS) data from Peter et al. (2018) included strain metadata, which we used to select heterozygous, diploid, and euploid strains. We assessed the quality of FASTQ files with FastQC v0.12.1 (Andrew 2010) and summarized FastQC reports with MultiQC v1.27 (Ewels et al. 2016). Based on the results, we filtered out libraries with fewer than 4 million reads (one strain) and a sequence duplication level exceeding 90% (3 strains). Altogether, of the 969 available RNA-seq-sequenced strains, we selected 295 for further analysis.

### Initial read mapping

RNA-seq reads were then mapped to the *S. cerevisiae* reference genome (R64-1-1) using HISAT2 v2.2.1 (Kim et al. 2019) and a modified GTF file downloaded from Ensembl. GTF modification included the removal of dubious ORFs (as defined in the SGD (*SGD_features.tab*, Saccharomyces Genome Database, downloaded on 19-10-2023) and the addition of UTR regions to the transcript. Transcript coordinates were adopted from Pelechano et al. (2013)—the longest transcripts covering an ORF in yeast grown in YPD or GAL were used. Duplicate reads were marked using GATK MarkDuplicates v4.1.9.0 (McKenna et al. 2010; DePristo et al. 2011; Van Der Auwera et al. 2013), and the resulting BAM files were sorted with SAMtools v1.9 (Danecek et al. 2021). Alignment metrics were summarized with MultiQC; based on its report, we excluded 11 strains with an alignment rate below 80%, resulting in a final dataset of 284 strains (Table S1).

### Selection of genes for variant calling and PTV analysis

We decided to exclude genes of low expression from variant calling and PTV selection. To identify such genes, we computed gene coverages using mosdepth v0.3.8 (Pedersen and Quinlan 2018). We then selected genes with moderate to high expression (read counts >20 in at least 40 samples) as a pragmatic threshold to ensure sufficient coverage for the detection of heterozygous variants and reliable estimation of allele-specific expression, while reducing noise from lowly expressed genes and enabling meaningful comparisons with population-level haplotype expression. Gene regions (as defined in the modified GTF described above) were then padded by 200 bp on both ends to ensure nearby regulatory variants were included. Finally, the overlapping regions were merged, and the resulting BED file was used for variant calling.

### Detection of genetic variants

RNA-seq alignments were processed using GATK SplitNCigarReads to handle reads spanning splice junctions. This step ensures that reads spanning intron-exon boundaries are correctly represented for subsequent downstream analyses. Variant calling was performed using FreeBayes v1.3.6 (Garrison and Marth 2012), with parameters optimized for the detection of low-frequency variants (*F* 0.05 -*z* 0.05 −use-duplicate-reads −0 -*Q* 15). Variants were called in regions defined in the reference BED file described above. Then, multiallelic sites were split with BCFtools v1.18 (bcftools norm -m -any) (Danecek et al. 2021). Variants were filtered to remove spanning deletions and reference-only sites using bcftools filter -e “ALT∼ “\*"’ | GT="ref"’, and heterozygous genotype calls were standardized to the 0/1 format. Variants were annotated with unique IDs (CHROM-POS-REF-ALT) using bcftools annotate with the −set-id parameter. VCF files were sorted and compressed using bcftools and tabix.

### Variant annotation

Variants were annotated using the Ensembl Variant Effect Predictor (VEP) v105.0 (McLaren et al. 2016) to predict their functional consequences. VEP was run in offline mode with a locally cached *S. cerevisiae* database. Next, we modified the CSQ annotation field by removing annotations for variants located in dubious open reading frames (ORFs), which are not expected to encode an expressed protein. The structured variant annotations from the VEP CSQ field were extracted into separate INFO fields using bcftools + split-vep. In addition, to retain only the most severe consequence per variant, a second bcftools + split-vep run was performed with the -s worst option. Complex variants were further decomposed into separate consecutive variant records using bcftools norm -a, and genotypes were adjusted to correspond to biallelic positions. Again, we split multiallelic positions using bcftools norm -m -any, and filtered out spanning deletions that may appear after splitting with bcftools filter -e “ALT∼ “\*”. These steps were performed to produce a standardized variant representation suitable for comparison with variants called from the WGS data (see below).

### Intersection with variants from WGS analysis and initial variant filtering

To establish a reliable set of variants for analysis, we validated the variants identified in RNA-seq data against the whole-genome sequencing variants reported by Peter et al. (2018). WGS variant data for 1,011 strains are publicly available as a single multisample GVCF file. We first filtered the dataset to retain only the 284 strains of interest (present in our filtered RNA-seq data). We split multiallelic positions (bcftools norm -m -any) and normalized INDELs (bcftools norm). To exclude unreliable variant calls, we applied two-step bcftools filter -e filtering: (i) sites where all genotypes were either missing or homozygous reference were excluded (‘COUNT(GT="mis”) + COUNT(GT="ref”) = 284'), (ii) variants with more than ∼10% missing genotype calls across strains were removed (‘COUNT(GT=“mis”) ≥ 28'). Then, variants were annotated using VEP, and annotations of variants located in dubious ORFs were removed. To identify putative protein-truncating variants, VEP annotations were split in 2 steps using the bcftools + split-vep plugin, with the -s worst option applied in the second run. Finally, we extracted strain-specific VCF files using bcftools view. Only genotypes carrying the alternate allele (GT="alt”) were retained. WGS variants were marked in RNA-seq VCF files with bcftools −mark-sites. All variants flagged as WGS variants were retained. Additionally, we kept highly confident variants called only in the RNA-seq data that met all of the following criteria: (i) sequencing depth (DP) > 10, (ii) alternate allele observations (AO) > 4, and (iii) quality score (QUAL) > 20. Filtering was performed using bcftools filter.

### Protein-truncating variants used in the analyses

We created lists of PTVs for each strain, including heterozygous variants common to the WGS and RNA-seq datasets. Variants were classified as PTVs based on the VEP Consequence field. We retained all variants whose consequence annotation contained at least one of the following Sequence Ontology terms: frameshift_variant, stop_gained, splice_donor_variant, or splice_acceptor_variant. We also retained compound consequence annotations involving these terms, resulting in 7 consequence categories. Then, we filtered variants to retain only genes with a single PTV mutation and without copy number variation (copy number data were obtained from Peter et al. (2018). After this step, 2 strains remained with no PTV variants, leaving 282 strains available for further analysis.

### Allele (haplotype)-specific transcript quantification

#### WGS reads mapping and variant phasing

To facilitate accurate allele-specific transcript quantification, we adopted a graph-based reference framework that reduces mapping bias and allows the integration of signals from nearby heterozygous variants. To this end, filtered VCF files were locally phased with WhatsHap v2.3 (Patterson et al. 2015). To achieve more complete phasing, we utilized previously generated RNA-seq along with WGS BAM files. To obtain the latter, paired-end WGS reads (Peter et al. 2018) were aligned to the *S. cerevisiae* reference genome (R64-1-1) using BWA-MEM v0.7.17-r1188 (Li 2013). Duplicate reads were marked using GATK MarkDuplicates, and the resulting BAM files were subsequently sorted and indexed with SAMtools. Homozygous genotypes in the output phased VCFs were standardized by converting 1/1 to 1|1.

#### Filtering VCF files based on phasing quality

Next, we checked the efficiency of the phasing process. Due to the specificity of RNA-seq data used (short reads of 75 bp, single-end sequencing), a considerable fraction of variants were not phased. For our purposes, local phasing within gene regions was sufficient; however, we had to exclude cases where phase sets could be switched within the same gene. Therefore, we decided to apply an additional filtering step. We retained one phase set of heterozygous variants per gene or, if no variants were phased, one heterozygous variant. PTV variants were always kept. For genes containing a PTV, we retained only the phase set encompassing the PTV (or the PTV alone if unphased) and discarded all other heterozygous variants. For control genes (ie genes without a PTV), we retained the phase set with the most variants or, when unphased, one randomly selected heterozygous variant. All homozygous variants were also retained. The resulting phased VCF files were later used to build indices for variation graphs and allele-specific read counting.

#### Vg—building and indexing graphs, mapping

We used vg v1.59.0 (Garrison et al. 2018) software to build and index strain-specific variation graphs, incorporating a set of genetic variants identified for each analyzed yeast strain. For each strain, sequencing reads were mapped to its corresponding graph to enable accurate alignment that accounts for known genetic variation. Using the vg autoindex command, we generated the necessary indices from the reference genome and phased VCFs. RNA-seq reads were aligned to the strain-specific spliced graph using vg mpmap, with prebuilt indices (.xg, .gcsa, and.dist) for each sample. Single-end FASTQ reads were mapped with RNA-seq-specific presets (-n RNA) and a read length setting appropriate for 75 bp reads (-l short). The fragment length and standard deviation were set to 200 and 50 bp (-I 200 -D 50), and sample identifiers were added via -N and -R. The alignments were output in GAMP multipath format, which is vg's equivalent of BAM, for downstream haplotype-aware quantification with rpvg.

#### Read count per haplotype—rpvg

Haplotype-specific read counts were obtained using rpvg (Sibbesen et al. 2023) with graph-based alignments. For each sample, spliced graph indices produced with vg autoindex −workflow rpvg were provided alongside GAMP alignments generated by vg mpmap. Quantification was performed in haplotype-transcript mode (-i haplotype-transcript) using a transcript-origin file to map graph nodes to transcripts. Fragment length parameters were set to a mean of 200 bp and a standard deviation of 50 bp, and strand-specific reads were handled according to the rf protocol. RPVG calculates the probability of each read originating from each transcript path, which is then used to calculate the probabilities of haplotype combinations. We decided to use the resulting rpvg-joint files, which include all possible diplotypes (pairs of haplotypes) and provide the joint probability for each haplotype pair. The possible outcomes are H1/H1, H1/H2, R1/H1, and R1/R1 diplotypes, where H1 and H2 stand for nonreference haplotype and R1 denote the reference haplotype.

#### Haplotypes decoding

Variant phasing was performed within individual genes, rather than continuously across the entire genomic region. Thus, it was essential to identify which haplotypes in the rpvg results carried PTV mutations. We first converted paths in the variation graphs into the FASTA files using the vg paths command with.gbwt (-g) and.xg (-x) indexes. The -R and -F options were used to include reference paths and export sequences in FASTA format. We filtered the FASTA files, retaining only sequences from genes that include PTV mutations. The resulting FASTA files were then reformatted into a sequence-per-line layout and split so that sequences corresponding to individual haplotypes were grouped using the grep -A1 command for H1, H2, and R1 haplotypes. We aligned the split FASTA files to the reference genome using both HISAT2 and Bowtie2 v2.4.2 (Langmead and Salzberg 2012) to maximize variant detection. Using 2 complementary alignment tools allowed us to capture a broader spectrum of variants, as each algorithm has different sensitivities and mapping strategies for handling sequence mismatches, INDELs, and spliced alignments. Variants were then called using FreeBayes with a minimum count of reads supporting an alternate allele of 1. Finally, we annotated variants using Variant Effect Predictor (VEP). We used the resulting VCF files to create a list of PTV mutations in each haplotype. At this stage, we refined the PTV variant lists to account for variants absent from the haplotype graph. Specifically, we excluded 13 cases corresponding to strain–variant combinations (one per strain, spanning nine genes).

### Validation of the graph-based method using WGS data

To control for bias introduced by the graph construction strategy, we replicated the RNA-seq transcript quantification procedures for the WGS dataset. Using vg software, we mapped WGS reads to the previously generated strain-specific spliced graph (which incorporated variants selected for the RNA-seq analysis). We then quantified reads mapped to each haplotype from the resulting graph-based alignments using rpvg.

### NMD analysis

#### Filtering rpvg output for NMD analyses

The following steps were done for both RNA-seq and WGS allele-specific read counts. From the rpvg-joint output files, we first removed homozygotes and diplotypes with zero read counts. Using previously generated lists of PTV mutations in specific haplotypes, we annotated each haplotype to indicate the presence of PTV. For this analysis, we used all heterozygous diplotypes with a haplotyping probability of one.

#### Modeling read count variation by PTV status

To assess whether PTV status (present vs. absent) triggers NMD, we fitted a generalized linear mixed model (GLMM) with a beta-binomial distribution and logit link. For each gene, we selected a single focal haplotype: for genes carrying a heterozygous PTV, we always selected the haplotype containing PTV; for all other genes (controls), we selected one haplotype—H1 for genes with R1/H1 diplotypes, or a randomly chosen haplotype for H1/H2 diplotypes. The model's response variable was specified as the number of reads of a focal haplotype (RC_focal_hapl) out of the total reads for the gene (RC_gene). PTV status (IS_PTV) was included as a fixed effect, and strain was modeled as a random intercept to account for strain-specific variability. Model formula:

cbind(RC_focal_hapl, RC_gene - RC_focal_hapl) ∼ IS_PTV + (1 | STRAIN)

Models were fitted using the glmmTMB package in R (v4.5.1).

#### Features associated with the NMD signal

Since NMD does not affect all transcripts equally, we examined whether there is a correlation between PTV allele fraction and: (i) PTC to transcript end distance, (ii) the length of the open reading frame (ORF), and (iii) PTV frequency in the population. Correlation analyses involving PTC-to-transcript-end distance were additionally performed separately for transcripts with 3′UTRs >300 nt and ≤300 nt. To assess whether differences in the observed correlation coefficients between these 2 groups could be explained by the reduced sample sizes after stratification, we performed a randomization test. In each of 1,000 iterations, 2 random subsets matching the sizes of the long- and short-3′UTR groups were sampled from the complete dataset, Spearman's correlation coefficients (*ρ*) were calculated for each subset, and the observed correlation coefficients were compared with the resulting null distributions. We also assessed whether variants with different predicted consequences exhibit differences in PTV allele fraction. We used a partial Spearman correlation to assess the relationship between ORF length and PTC-to-end distance, controlling for PTC-to-end distance, and a standard Spearman correlation to assess the relationship between PTC-to-end distance and ORF length. Transcript lengths were retrieved from Ensembl BioMart (Ensembl release 114) (Dyer et al. 2025). For each PTV, we identified the position of the premature stop codon from the HGVSp field in the VCF files. We calculated its distance from the transcript end as the absolute difference between the transcript length and the PTC position. We compared PTV fractions across variant consequence categories annotated by VEP using the Kruskal–Wallis test, followed by pairwise Wilcoxon rank-sum comparisons with multiple testing correction. Finally, we examined the relationship between the population-level distribution of PTV alleles (calculated as the number of PTV copies divided by twice the total number of strains) and the PTV allele fractions.

### Compensation analysis

#### Generation of read count matrices

We filtered the rpvg-joint output files so that, for each gene, the diplotype with the highest haplotyping probability was selected. If multiple diplotypes share the same haplotyping probability, we chose either one at random or the diplotype with nonzero counts. Then, we recalculated the read counts by dividing them by the haplotyping probability. We prepared 2 types of matrices for differential gene expression analysis (DGE): (i) read counts per gene, and (ii) read counts per haplotype. For the haplotype counts, we selected the haplotype without a PTV in heterozygous PTV genotypes. In other heterozygotes, we selected a reference allele, if present, or a random nonreference allele. In homozygotes, we chose one random haplotype.

#### Differential gene expression analysis for PTV genes

We aimed to investigate whether there is compensatory upregulation in the healthy allele of heterozygous genes carrying a PTV. To this end, we compared the expression of the healthy allele in the heterozygous PTV gene with that of a selected haplotype (described above) of the same gene across the set of control strains. Here, we used a per-haplotype read count matrix. We performed DGE analysis using edgeR v4.6.3 (Chen et al. 2025) with TMM normalization to account for differences in library size and compositional bias between samples. Thus, each PTV gene in a given strain was tested separately against a set of control strains lacking PTVs and copy-number variations in the tested gene.

#### Differential gene expression analysis for PTV genes’ paralogs

For each strain, we generated a list of paralogs of PTV genes (paralog data were retrieved from Ensembl BioMart, Ensembl release 114). From the paralog lists, we excluded any genes that were also present in the PTV list for the same strain. For each paralog gene, we performed a similar DGE analysis using total gene read counts. Here, control strains were free of PTVs and copy number variations in the pair of genes—PTV and its paralog.

#### Coefficient of variation and mean expression

To assess variability of PTV gene expression across strains, we calculated the coefficient of variation (CV). We used TMM-normalized read counts for 282 strains across 5,344 genes to compute the mean and standard deviation of expression per gene, and then obtained the CV by dividing the standard deviation by the mean. We assessed the correlation between mean logFC per gene and (i) mean expression using Spearman correlation, and (ii) the coefficient of variation using partial Spearman correlation while controlling for mean expression.

#### Randomization test

To assess whether the observed compensation pattern associated with PTVs and their paralogs could arise by chance, we performed a randomization test with 1,000 independent iterations using haplotype- and gene-level read counts, respectively. In each iteration, for each strain, we randomly selected a set of genes and corresponding control strains matching the number of tested PTV genes and controls, and repeated the DGE analysis. We then tested the difference between the observed median logFC of the PTVs/paralogs and the median logFC distribution obtained from random draws. To do this, we computed an empirical *P*-value, defined as the proportion of randomization-derived medians less than or equal to the observed PTVs/paralogs median, with a + 1 correction.

#### Compensation in genes with low-expression variability

To assess whether genes with more stable expression between strains exhibit a distinct compensatory response, we repeated the compensation analysis on a group of genes with the lowest expression variability across strains. Specifically, we selected the lowest quartile (25%) of PTV genes ranked by coefficient of variation (CV) and DGE analysis restricted to this subset. To evaluate statistical significance, we used a randomization-based approach (*n* = 1,000). For each strain, permutation results were filtered to (i) match the number of genes tested in the corresponding PTV differential expression analysis and (ii) include only genes with a CV lower than or equal to the maximum CV observed among the selected lowest-quartile PTV genes. In addition, genes were ranked by CV in ascending order, and cumulative subsets were constructed by progressively increasing the inclusion threshold in 5% increments. For each subset, we calculated the median log fold-change (logFC).

## Supplementary Material

msag219_Supplementary_Data

## Data Availability

Data and code supporting the findings of this study are publicly available in the GitHub repository (https://github.com/MarzenaMarszalek/Yeast-Compensation) and archived on Zenodo (DOI: https://doi.org/10.5281/zenodo.21340969). Several main tables are provided as Supplementary Materials.

## References

[msag219-B1] Amrani N et al A faux 3′-UTR promotes aberrant termination and triggers nonsense- mediated mRNA decay. Nature. 2004:432:112–118. 10.1038/nature03060. 15525991

[msag219-B2] Andjus S et al Pervasive translation of Xrn1-sensitive unstable long noncoding RNAs in yeast. RNA. 2024:30:662–679. 10.1261/rna.079903.123. 38443115 PMC11098462

[msag219-B3] Andrew S . A quality control tool for high throughput sequence data. 2010. http://www.bioinformatics.babraham.ac.uk/projects/fastqc/. Accessed June 2024.

[msag219-B4] Arribere JA, Fire AZ. Nonsense mRNA suppression via nonstop decay. eLife. 2018:7:e33292. 10.7554/eLife.33292. 29309033 PMC5777819

[msag219-B5] Caudal É et al Pan-transcriptome reveals a large accessory genome contribution to gene expression variation in yeast. Nat Genet. 2024:56:1278–1287. 10.1038/s41588-024-01769-9. 38778243 PMC11176082

[msag219-B6] Celik A, Baker R, He F, Jacobson A. High-resolution profiling of NMD targets in yeast reveals translational fidelity as a basis for substrate selection. RNA. 2017:23:735–748. 10.1261/rna.060541.116. 28209632 PMC5393182

[msag219-B7] Chen Y, Chen L, Lun ATL, Baldoni PL, Smyth GK. Edger v4: powerful differential analysis of sequencing data with expanded functionality and improved support for small counts and larger datasets. Nucleic Acids Res. 2025:53:gkaf018. 10.1093/nar/gkaf018. 39844453 PMC11754124

[msag219-B8] Danecek P et al Twelve years of SAMtools and BCFtools. GigaScience. 2021:10:giab008. 10.1093/gigascience/giab008. PMC7931819 33590861

[msag219-B9] Decourty L et al Long open Reading frame transcripts Escape nonsense-mediated mRNA decay in yeast. Cell Rep. 2014:6:593–598. 10.1016/j.celrep.2014.01.025. 24529707

[msag219-B10] DeLuna A, Springer M, Kirschner MW, Kishony R. Need-based up-regulation of protein levels in response to deletion of their duplicate genes. PLoS Biol. 2010:8:e1000347. 10.1371/journal.pbio.1000347. 20361019 PMC2846854

[msag219-B11] De Nadal E, Ammerer G, Posas F. Controlling gene expression in response to stress. Nat Rev Genet. 2011:12:833–845. 10.1038/nrg3055. 22048664

[msag219-B12] DePristo MA et al A framework for variation discovery and genotyping using next-generation DNA sequencing data. Nat Genet. 2011:43:491–498. 10.1038/ng.806. 21478889 PMC3083463

[msag219-B13] Dyer SC et al Ensembl 2025. Nucleic Acids Res. 2025:53:D948–D957. 10.1093/nar/gkae1071. 39656687 PMC11701638

[msag219-B14] El-Brolosy MA et al Genetic compensation triggered by mutant mRNA degradation. Nature. 2019:568:193–197. 10.1038/s41586-019-1064-z. 30944477 PMC6707827

[msag219-B15] El-Brolosy MA et al Mechanisms linking cytoplasmic decay of translation-defective mRNA to transcriptional adaptation. Science. 2026:391:eaea1272. 10.1126/science.aea1272. 41678638 PMC13286266

[msag219-B16] Evans C, Hardin J, Stoebel DM. Selecting between-sample RNA-Seq normalization methods from the perspective of their assumptions. Brief Bioinform. 2018:19:776–792. 10.1093/bib/bbx008. 28334202 PMC6171491

[msag219-B17] Ewels P, Magnusson M, Lundin S, Käller M. MultiQC: summarize analysis results for multiple tools and samples in a single report. Bioinformatics. 2016:32:3047–3048. 10.1093/bioinformatics/btw354. 27312411 PMC5039924

[msag219-B18] Falcucci L et al Transcriptional adaptation upregulates utrophin in Duchenne muscular dystrophy. Nature. 2025:639:493–502. 10.1038/s41586-024-08539-x. 39939773 PMC11903304

[msag219-B19] García-Martínez J et al The total mRNA concentration buffering system in yeast is global rather than gene-specific. RNA. 2021:27:1281–1290. 10.1261/rna.078774.121. 34272303 PMC8456998

[msag219-B20] Garrison E et al Variation graph toolkit improves read mapping by representing genetic variation in the reference. Nat Biotechnol. 2018:36:875–879. 10.1038/nbt.4227. 30125266 PMC6126949

[msag219-B21] Garrison E, Marth G. Haplotype-based variant detection from short-read sequencing. arXiv 3907. 10.48550/arXiv.1207.3907, 17 July 2012, preprint: not peer reviewed.

[msag219-B22] Gasch AP et al Genomic expression programs in the response of yeast cells to environmental changes. Mol Biol Cell. 2000:11:4241–4257. 10.1091/mbc.11.12.4241. 11102521 PMC15070

[msag219-B23] Gu Z et al Role of duplicate genes in genetic robustness against null mutations. Nature. 2003:421:63–66. 10.1038/nature01198. 12511954

[msag219-B24] Haldane JBS . A note on Fisher's theory of the origin of dominance, and on a correlation between dominance and linkage. Am Nat. 1930:64:87–90. 10.1086/280299.

[msag219-B25] He F, Jacobson A. Nonsense-mediated mRNA decay: degradation of defective transcripts is only part of the story. Annu Rev Genet. 2015:49:339–366. 10.1146/annurev-genet-112414-054639. 26436458 PMC4837945

[msag219-B26] Hill MS, Vande Zande P, Wittkopp PJ. Molecular and evolutionary processes generating variation in gene expression. Nat Rev Genet. 2021:22:203–215. 10.1038/s41576-020-00304-w. 33268840 PMC7981258

[msag219-B27] Hose J et al Dosage compensation can buffer copy-number variation in wild yeast. eLife. 2015:4:e05462. 10.7554/eLife.05462. 25955966 PMC4448642

[msag219-B28] Kacser H, Burns JA. The molecular basis of dominance. Genetics. 1981:97:639–666. 10.1093/genetics/97.3-4.639. 7297851 PMC1214416

[msag219-B29] Kafri R, Bar-Even A, Pilpel Y. Transcription control reprogramming in genetic backup circuits. Nat Genet. 2005:37:295–299. 10.1038/ng1523. 15723064

[msag219-B30] Kebaara BW, Atkin AL. Long 3′-UTRs target wild-type mRNAs for nonsense-mediated mRNA decay in *Saccharomyces cerevisiae*. Nucleic Acids Res. 2009:37:2771–2778. 10.1093/nar/gkp146. 19270062 PMC2685090

[msag219-B31] Khaitovich P et al A neutral model of transcriptome evolution. PLoS Biol. 2004:2:e132. 10.1371/journal.pbio.0020132. 15138501 PMC406393

[msag219-B32] Kim D, Paggi JM, Park C, Bennett C, Salzberg SL. Graph-based genome alignment and genotyping with HISAT2 and HISAT-genotype. Nat Biotechnol. 2019:37:907–915. 10.1038/s41587-019-0201-4. 31375807 PMC7605509

[msag219-B33] Kovács K et al Suboptimal global transcriptional response increases the harmful effects of loss-of-function mutations. Mol Biol Evol. 2021:38:1137–1150. 10.1093/molbev/msaa280. 33306797 PMC7947755

[msag219-B34] Langmead B, Salzberg SL. Fast gapped-read alignment with bowtie 2. Nat Methods. 2012:9:357–359. 10.1038/nmeth.1923. 22388286 PMC3322381

[msag219-B35] Li H . Aligning sequence reads, clone sequences and assembly contigs with BWA-MEM. arXiv 3997. 10.48550/arXiv.1303.3997, 16 March 2013, preprint: not peer reviewed.

[msag219-B36] Ma Z et al PTC-bearing mRNA elicits a genetic compensation response via Upf3a and COMPASS components. Nature. 2019:568:259–263. 10.1038/s41586-019-1057-y. 30944473

[msag219-B37] MacArthur DG et al A systematic survey of loss-of-function variants in human protein-coding genes. Science. 2012:335:823–828. 10.1126/science.1215040. 22344438 PMC3299548

[msag219-B38] Malabat C, Feuerbach F, Ma L, Saveanu C, Jacquier A. Quality control of transcription start site selection by nonsense-mediated-mRNA decay. eLife. 2015:4:e06722. 10.7554/eLife.06722. 25905671 PMC4434318

[msag219-B39] McAnally AA, Yampolsky LY. Widespread transcriptional autosomal dosage compensation in Drosophila correlates with gene expression level. Genome Biol Evol. 2009:2:44–52. 10.1093/gbe/evp054. 20333221 PMC2839349

[msag219-B40] McKenna A et al The genome analysis toolkit: a MapReduce framework for analyzing next-generation DNA sequencing data. Genome Res. 2010:20:1297–1303. 10.1101/gr.107524.110. 20644199 PMC2928508

[msag219-B41] McLaren W et al The ensembl variant effect predictor. Genome Biol. 2016:17:122. 10.1186/s13059-016-0974-4. 27268795 PMC4893825

[msag219-B42] Mellis IA, Melzer ME, Bodkin N, Goyal Y. Prevalence of and gene regulatory constraints on transcriptional adaptation in single cells. Genome Biol. 2024:25:217. 10.1186/s13059-024-03351-2. 39135102 PMC11320884

[msag219-B43] Muenzner J et al Natural proteome diversity links aneuploidy tolerance to protein turnover. Nature. 2024:630:149–157. 10.1038/s41586-024-07442-9. 38778096 PMC11153158

[msag219-B44] Normanly J, Bartelt B. Redundancy as a way of life - IAA metabolism. Curr Opin Plant Biol. 1999:2:207–213. 10.1016/S1369-5266(99)80037-5. 10375566

[msag219-B45] Patterson M et al WhatsHap: weighted haplotype assembly for future-generation sequencing reads. J Comput Biol. 2015:22:498–509. 10.1089/cmb.2014.0157. 25658651

[msag219-B46] Pedersen BS, Quinlan AR. Mosdepth: quick coverage calculation for genomes and exomes. Bioinformatics. 2018:34:867–868. 10.1093/bioinformatics/btx699. 29096012 PMC6030888

[msag219-B47] Pelechano V, Wei W, Steinmetz LM. Extensive transcriptional heterogeneity revealed by isoform profiling. Nature. 2013:497:127–131. 10.1038/nature12121. 23615609 PMC3705217

[msag219-B48] Peter J et al Genome evolution across 1,011 Saccharomyces cerevisiae isolates. Nature. 2018:556:339–344. 10.1038/s41586-018-0030-5. 29643504 PMC6784862

[msag219-B49] Rivas MA et al Human genomics. Effect of predicted protein-truncating genetic variants on the human transcriptome. Science. 2015:348:666–669. 10.1126/science.1261877. 25954003 PMC4537935

[msag219-B50] Robinson MD, Oshlack A. A scaling normalization method for differential expression analysis of RNA-seq data. Genome Biol. 2010:11:R25. 10.1186/gb-2010-11-3-r25. 20196867 PMC2864565

[msag219-B51] Rossi A et al Genetic compensation induced by deleterious mutations but not gene knockdowns. Nature. 2015:524:230–233. 10.1038/nature14580. 26168398

[msag219-B52] Ruiz-Gutierrez N, Graille M, Le Hir H, Saveanu C. Biochemical insights into the conserved interactions of NMD factors from budding yeast to humans. J Mol Biol. 2026:169751. 10.1016/j.jmb.2026.169751. 41819420

[msag219-B53] Serobyan V et al Transcriptional adaptation in *Caenorhabditis elegans*. eLife. 2020:9:e50014. 10.7554/eLife.50014. 31951195 PMC6968918

[msag219-B54] Sheltzer JM, Torres EM, Dunham MJ, Amon A. Transcriptional consequences of aneuploidy. Proc Natl Acad Sci U S A. 2012:109:12644–12649. 10.1073/pnas.1209227109. 22802626 PMC3411958

[msag219-B55] Sibbesen JA et al Haplotype-aware pantranscriptome analyses using spliced pangenome graphs. Nat Methods. 2023:20:239–247. 10.1038/s41592-022-01731-9. 36646895

[msag219-B56] Siddiq MA, Wittkopp PJ. Mechanisms of regulatory evolution in yeast. Curr Opin Genet Dev. 2022:77:101998. 10.1016/j.gde.2022.101998. 36220001 PMC10117219

[msag219-B57] Smith JE et al Translation of small open Reading frames within unannotated RNA transcripts in *Saccharomyces cerevisiae*. Cell Rep. 2014:7:1858–1866. 10.1016/j.celrep.2014.05.023. 24931603 PMC4105149

[msag219-B58] Springer M, Weissman JS, Kirschner MW. A general lack of compensation for gene dosage in yeast. Mol Syst Biol. 2010:6:368. 10.1038/msb.2010.19. 20461075 PMC2890323

[msag219-B59] Teng X et al Genome-wide consequences of deleting any single gene. Mol Cell. 2013:52:485–494. 10.1016/j.molcel.2013.09.026. 24211263 PMC3975072

[msag219-B60] Teran NA et al Nonsense-mediated decay is highly stable across individuals and tissues. Am J Hum Genet. 2021:108:1401–1408. 10.1016/j.ajhg.2021.06.008. 34216550 PMC8387471

[msag219-B61] Torres EM, Springer M, Amon A. No current evidence for widespread dosage compensation in *S. cerevisiae*. eLife. 2016:5:e10996. 10.7554/eLife.10996. 26949255 PMC4798953

[msag219-B62] Tutaj H, Tomala K, Pirog A, Marszałek M, Korona R. Extreme positive epistasis for fitness in monosomic yeast strains. eLife. 2024:12:RP87455. 10.7554/eLife.87455. 39417696 PMC11486488

[msag219-B63] Van Der Auwera GA et al From FastQ data to high confidence variant calls: the genome analysis toolkit best practices pipeline. Curr Protoc Bioinforma. 2013:43:11.10.1–11.10.33. 10.1002/0471250953.bi1110s43. PMC4243306 25431634

[msag219-B64] Vande Zande P, Siddiq MA, Hodgins-Davis A, Kim L, Wittkopp PJ. Active compensation for changes in TDH3 expression mediated by direct regulators of TDH3 in *Saccharomyces cerevisiae*. PLoS Genet. 2023:19:e1011078. 10.1371/journal.pgen.1011078. 38091349 PMC10752532

[msag219-B65] Wery M et al Nonsense-mediated decay restricts LncRNA levels in yeast unless blocked by double-stranded RNA structure. Mol Cell. 2016:61:379–392. 10.1016/j.molcel.2015.12.020. 26805575 PMC4747904

[msag219-B66] Wery M et al The role of nonsense-mediated mRNA decay in restricting long noncoding RNA expression has been conserved in RNAi-capable budding yeast. RNA. 2025:31:1886–1900. 10.1261/rna.080458.125. 41027714 PMC12621597

[msag219-B67] Yang J-R, Maclean CJ, Park C, Zhao H, Zhang J. Intra and interspecific variations of gene expression levels in yeast are largely neutral: (nei lecture, SMBE 2016, gold coast). Mol Biol Evol. 2017:34:2125–2139. 10.1093/molbev/msx171. 28575451 PMC5850415

