# Supplementary material for: Signatures of nonsense-mediated mRNA decay but no evidence for transcriptional adaptation associated with protein-truncating variants in wild yeast diploids
Source: Mol Biol Evol. 2026 Aug 21;43(9):msag219. doi: 10.1093/molbev/msag219 (PMC13537020; doi:10.1093/molbev/msag219)
Supplement: Supplementary file 1 [file msag219_supplementary_data.zip › Marszalek_Supplementary_Materials.pdf]

Supplementary Materials

Signatures of nonsense-mediated mRNA decay but no evidence for transcriptional adaptation associated with protein-truncating variants in wild yeast diploids

Marzena Marszałek , Wiesław Babik, Ryszard Korona, Katarzyna Tomala

Comparison between rpvg and FreeBayes estimates of allele fractions 2
Nonlinear relationship between NMD efficiency and 3' UTR length.................................... 5
Variant Effect Predictor consequence categories... 7

Comparison between rpvg and FreeBayes estimates of allele fractions

Differences in allelic fractions derived from linear (FreeBayes) and graph-based (rpvg) workflows might have three possible sources. First, linear mapping may fail to align some reads, while others may receive lower mapping quality scores. This effect should be particularly pronounced for reads containing INDELs or multiple mismatches, as lower alignment scores reduce the difference between the best and second-best alignments, thereby decreasing mapping confidence (mapping quality). Second, the standard FreeBayes filtering parameters used in our variant-calling pipeline (-0 option) exclude reads with low mapping or base quality. While appropriate for variant calling, these filters may lead to an underestimation of mutant allele fractions. Third, rpvg incorporates not only reads directly overlapping a variant but also reads that are phased with the variant. The first two effects are manifestations of reference mapping bias, whereas the third reflects rpvg's ability to leverage haplotype information when estimating allele fractions.

To explore these possibilities, we compared the agreement between FreeBayes and rpvg separately for phased and unphased variants, as well as for SNPs and INDELs (Figure S1). We also compared gene-level counts obtained with featureCounts (linear-reference alignment-based counts without mapping quality filtering) with summed rpvg transcript counts for a subset of 10 strains (AAP, ALH, ASP, BHI, BGQ, BHD, CIS, CPA, CQL, CQP).

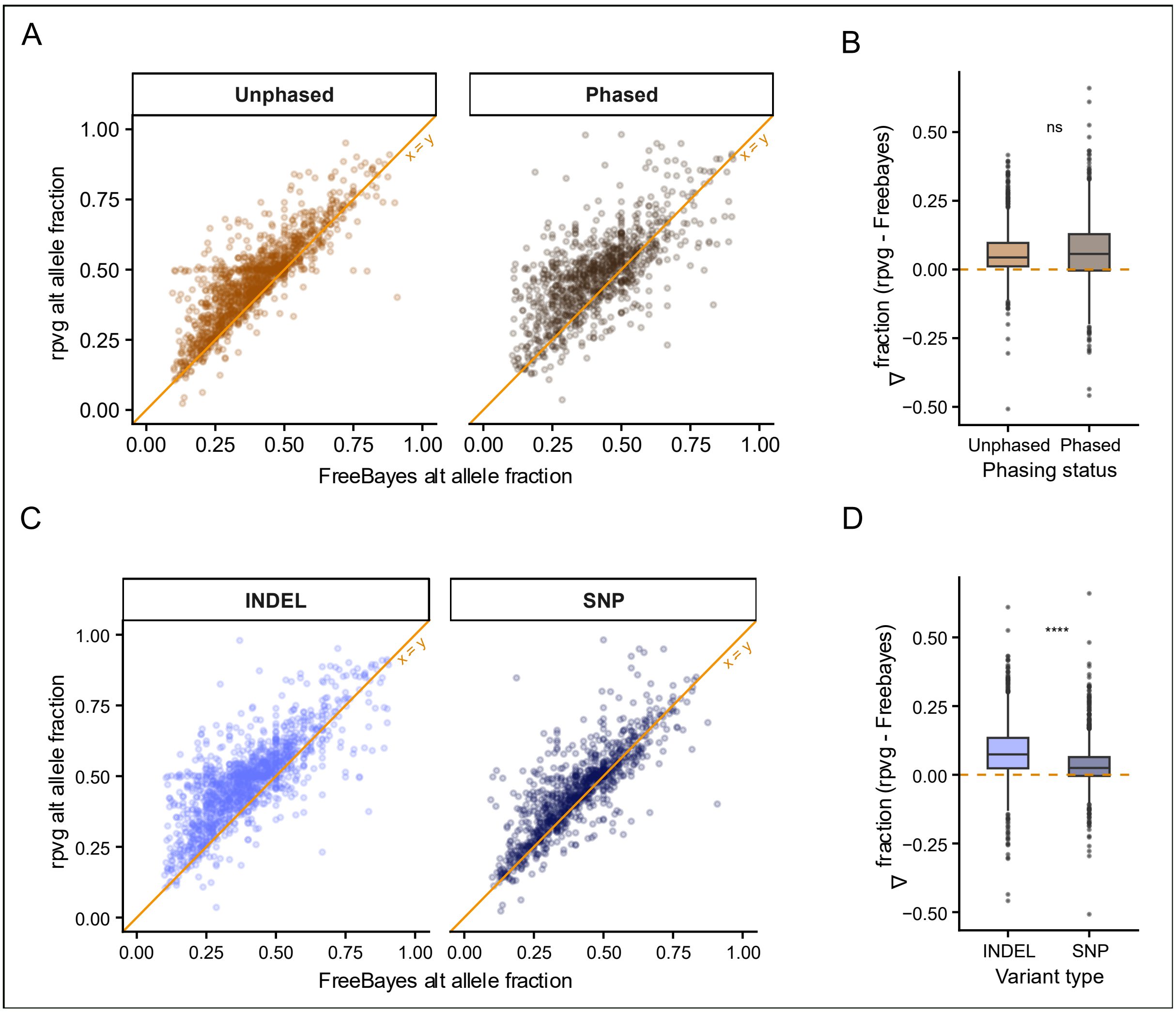

Figure S1 | (A) Correlation between alternative allele fractions estimated by rpvg and FreeBayes, shown separately for phased and unphased variants. **(B)** Differences between rpvg and FreeBayes alternative allele fraction estimates (rpvg − FreeBayes) for phased and unphased variants. **(C)** Correlation between alternative allele fractions estimated by rpvg and FreeBayes, shown separately for INDELs and SNPs (complex variants are included in the SNP group). **(D)** Differences between rpvg and FreeBayes alternative allele fraction estimates (rpvg − FreeBayes) for INDELs and SNPs.

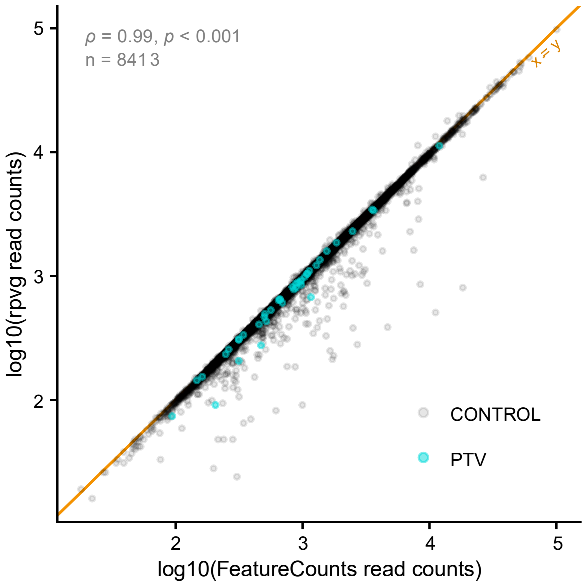

Figure S2 | Correlation of gene-level read counts from linear (featureCounts applied to HISAT2 alignments) and graph-based (rpvg applied to vg-based alignments) workflows. The analysis was restricted to a subset of 10 representative strains, and genes included in the NMD analysis (HP = 1; genes harboring heterozygous mutations in the respective strains).

The results suggest that phasing may have a minor effect, reflected in the greater dispersion of points between rpvg and FreeBayes for phased variants (Figure S1A), although the differences between rpvg and FreeBayes estimates (rpvg − FreeBayes) do not differ significantly between phased and unphased variants (Wilcoxon rank-sum test: W = 613,264, p = 0.256; Figure S1B). More importantly, the observed differences between the tools are consistent with reference mapping bias. However, they are unlikely to result primarily from widespread alignment failure, as many affected reads appear to align successfully. Instead, the differences may arise from FreeBayes preferentially filtering lower-mapping-quality reads. This interpretation is supported by the observation that differences between rpvg and FreeBayes estimates (rpvg − FreeBayes) are significantly larger for INDELs than for SNPs (Wilcoxon rank-sum test: W = 847,370, $p < 2.2 \times 10^{-16}$; Figure S1C-D). Moreover, gene-level counts obtained with rpvg and featureCounts are in excellent agreement (Spearman's $\rho = 0.99$), with the vast majority of genes lying close to the identity line (Figure S2). This indicates

that most reads are successfully aligned and assigned to genes, arguing against widespread alignment failure and supporting the interpretation that differences in read assignment confidence, such as those reflected by mapping quality, contribute to the observed differences. The small number of genes carrying PTVs (n = 41) did not appear to deviate from this pattern, although the sample size limits interpretation.

Nonlinear relationship between NMD efficiency and 3′ UTR length

According to the threshold model, the correlation between 3′ UTR length and the fraction of the PTV-bearing transcript should disappear after stratifying the data at the 300-nt threshold – transcripts with shorter 3’ UTRs should exhibit an allelic fraction close to 0.5. This is the pattern observed in our data. After stratification, the correlation is substantially reduced and is no longer statistically significant for transcripts with 3’ UTRs ≤300 nt, while it is reversed for transcripts with 3’ UTRs >300 nt (Figure S3B). The median allelic fraction in the short-UTR group shifts toward 0.5, although it remains significantly below this value (Wilcoxon signed-rank test, median = 0.484, n = 735, p < 0.001; Figure S3B). Importantly, the long-UTR group exhibits significantly lower allelic fractions than the short-UTR group (Wilcoxon rank-sum test, W = 429954, $p < 2.2 \times 10^{-16}$; Figure S3B). Randomization tests confirmed that the smaller sample sizes do not explain the observed reductions in correlation coefficients in the stratified analyses ($p < 0.001$ for both groups; Figure S4).

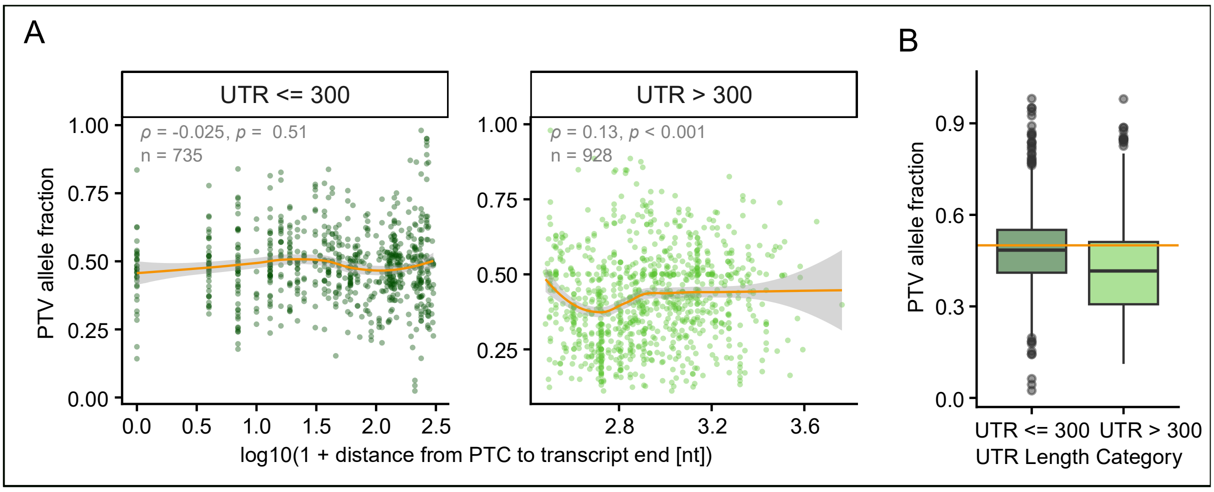

Figure S3 | (A) Correlation between 3' UTR length and PTV allele fraction for transcripts with long (>300 nt) and short (≤300 nt) 3' UTRs. Spearman's ρ values are shown. Fitted lines represent LOESS smooths. **(B)** Differences in PTV allele fractions between the long- and short-UTR groups. The orange horizontal line indicates an allelic fraction of 0.5.

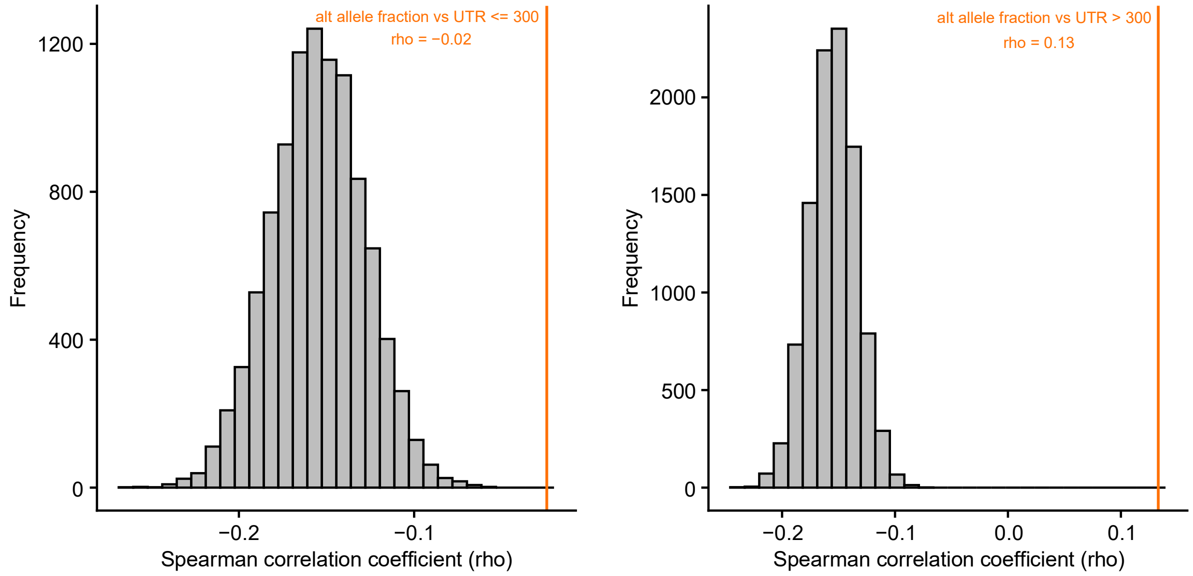

Figure S4 | Distribution of Spearman's rho (ρ) values obtained from 10,000 random draws from the complete dataset for 3' UTR length. In each iteration, two random subsets matching the sizes of the short- and long-3' UTR groups were sampled. The left and right panels show the distributions for the short- and long-3' UTR group sample sizes, respectively.

Variant Effect Predictor consequence categories

The VEP consequence categories included in our analysis differ in the confidence with which they can be expected to introduce a premature termination codon and cause loss of function. Variants annotated as ***stop_gained*** or ***stop_gained&frameshift_variant*** represent the most confident classes, as they directly introduce a premature termination codon. ***frameshift_variant*** typically results in a premature stop codon, as indicated by the HGVSp annotation (approximately 75% of cases in our dataset), but may also lead to protein elongation or transcripts lacking an in-frame stop codon and therefore subject to degradation. ***frameshift_variant&start_lost*** may lead to the use of alternative downstream start codons, potentially resulting in N-terminally truncated proteins, translation in an alternative reading frame, or loss of translation. ***frameshift_variant&start_lost&start_retained_variant*** denotes cases in which an indel disrupts the start codon while an alternative start codon is predicted at the same position, potentially resulting in translation in an alternative reading frame or loss of translation. ***frameshift_variant&stop_lost*** reflects overlapping annotations involving both a frameshift and loss of the canonical stop codon, which may result in elongated proteins or transcripts lacking a proper termination signal and therefore subject to degradation. Finally, ***frameshift_variant&stop_retained_variant*** may result in a short frameshift tract followed by restoration of the original stop codon, or may have no detectable functional consequence.
